# Fe_3_O_4_ Nanoparticles: Structures, Synthesis, Magnetic Properties, Surface Functionalization, and Emerging Applications

**DOI:** 10.3390/app112311301

**Published:** 2021-11-29

**Authors:** Minh Dang Nguyen, Hung-Vu Tran, Shoujun Xu, T. Randall Lee

**Affiliations:** Department of Chemistry and the Texas Center for Superconductivity, University of Houston, 4800 Calhoun Road, Houston, TX 77204-5003, USA;

**Keywords:** Fe_3_O_4_ nanoparticles, magnetic properties, core-shell structures, nanocomposites, surface functionalization, biomedical applications, biosensing, environmental applications, energy storage

## Abstract

Magnetite (Fe_3_O_4_) nanoparticles (NPs) are attractive nanomaterials in the field of material science, chemistry, and physics because of their valuable properties, such as soft ferromagnetism, half-metallicity, and biocompatibility. Various structures of Fe_3_O_4_ NPs with different sizes, geometries, and nanoarchitectures have been synthesized, and the related properties have been studied with targets in multiple fields of applications, including biomedical devices, electronic devices, environmental solutions, and energy applications. Tailoring the sizes, geometries, magnetic properties, and functionalities is an important task that determines the performance of Fe_3_O_4_ NPs in many applications. Therefore, this review focuses on the crucial aspects of Fe_3_O_4_ NPs, including structures, synthesis, magnetic properties, and strategies for functionalization, which jointly determine the application performance of various Fe_3_O_4_ NP-based systems. We first summarize the recent advances in the synthesis of magnetite NPs with different sizes, morphologies, and magnetic properties. We also highlight the importance of synthetic factors in controlling the structures and properties of NPs, such as the uniformity of sizes, morphology, surfaces, and magnetic properties. Moreover, emerging applications using Fe_3_O_4_ NPs and their functionalized nanostructures are also highlighted with a focus on applications in biomedical technologies, biosensing, environmental remedies for water treatment, and energy storage and conversion devices.

## 1. Introduction

**Figure 1.**
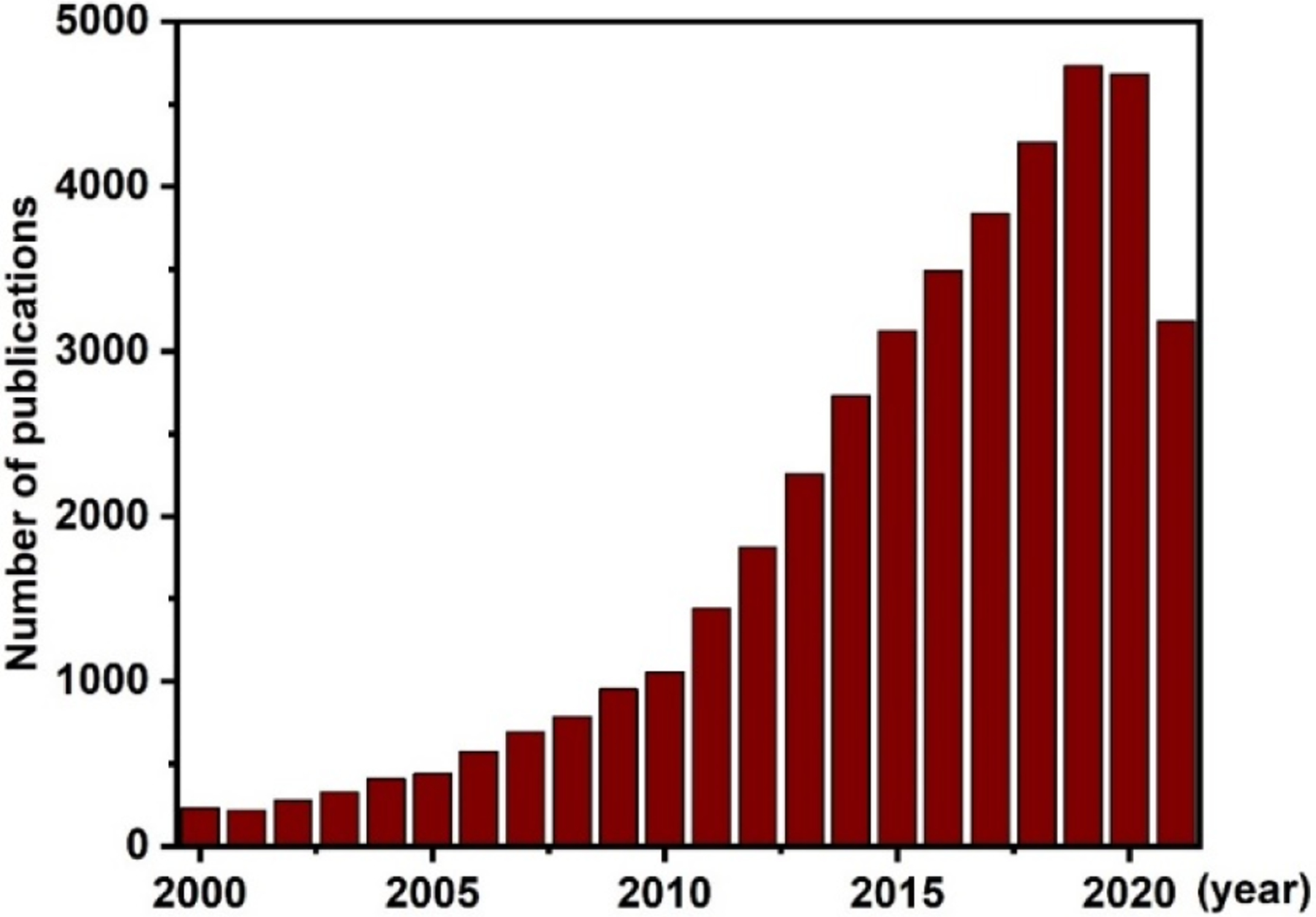
Numbers of articles with the keyword “Fe_3_O_4_” published in the last 20 years. Data originated from the Web of Science. (Data from 2021 were collected until September 2021).

Magnetic nanoparticles (MNPs) are an interesting class of nanomaterials that have been extensively explored for use in many technological applications [1–5]. MNPs have been utilized in sensing technologies, memory storage devices, magnetic separation, magnetic labeling, and catalytic processes [6–9]. In biomedical applications, MNPs have been used to induce heating for hyperthermia treatments, to provide contrast effects for magnetic imaging, and for the remote control of targeted drug delivery [10,11]. Among magnetic materials, iron oxide NPs are promising nanomaterials due to their great biocompatibility [12,13]. The biocompatibility of iron oxide NPs is the main driving force of substantial research efforts to commercialize these NPs for advanced medical technology applications [14]. Although numerous iron oxides are known, the term “iron oxides” typically refers to three types: Fe_3_O_4_ (magnetite), α-Fe_2_O_3_ (hematite), and γ-Fe_2_O_3_ (maghemite) [15]. Among all iron oxides, Fe_3_O_4_ has attracted more attention due to its superior magnetic properties. In the last two decades, research on Fe_3_O_4_ NPs has achieved remarkable progress in not only the synthesis of homogeneous core magnetic Fe_3_O_4_ NPs but also the preparation of advanced nanoarchitectures (core–shell, composites, functionalized surfaces, etc.) and the application of these nanomaterials in various fields [16–19]. According to the Web of Science, more than 41,000 research papers with the keyword “Fe_3_O_4_” have been published in the last 20 years, as shown in Figure 1. In the last 10 years, the number of these publications has increased drastically, with several hundred papers published per year. These numbers reflect the great attention given to Fe_3_O_4_ nanomaterials by the research community in both fundamental studies and applied science.

Fe_3_O_4_ has attracted more attention than other iron oxides or ferrite spinel oxides (MFe_2_O_4_ with M = Co, Ni, Mg, etc.) because of its superior magnetic properties, electronic conductivity, and biocompatibility. The magnetic properties of Fe_3_O_4_ can be explained by its crystal structure. Magnetite has a cubic inverse spinel crystal structure consisting of Fe^2+^ cations occupying 25% of the octahedral interstitial sites and Fe^3+^ cations occupying 25% of the octahedral sites and 12.5% of the tetrahedral sites, and thirty-two O^2−^ anions in its unit cell [20,21]. The magnetic moments of Fe^3+^ and Fe^2+^ cations in octahedral holes are coupled ferromagnetically. However, the Fe^3+^ ions in tetrahedral sites possess magnetic dipoles in the reverse direction of the Fe^3+^ ions in the octahedral sites. Therefore, Fe_3_O_4_ is a ferrimagnetic material with high saturation magnetization (M_S_) and low coercivity (H_C_) due to the antiferromagnetically coupled Fe^3+^ cations in tetrahedral and octahedral sites of its crystal structure. Sometimes, the term ferromagnetic properties or ferromagnetism is also used for Fe_3_O_4,_ which indicates that it exhibits magnetic properties in the absence of a magnetic field. Regarding other iron oxide phases, maghemite is also a ferrimagnetic material, and hematite is considered a weak ferromagnetic material. The maximum values of saturation magnetization (M_S_) for magnetite (Fe_3_O_4_) and maghemite (γ-Fe_2_O_3_) are 98 emu/g and 82 emu/g, respectively [10], whereas the values for saturation magnetization of hematite (α-Fe_2_O_3_) are usually modest and reported to be approximately 2 to 4 emu/g [22–24]. In the case of spinel ferrite MFe_2_O_4_, the maximum saturation magnetization values for CoFe_2_O_4_, NiFe_2_O_4_, and MgFe_2_O_4_ are 94 emu/g, 56 emu/g, and 31 emu/g, respectively [10]. Regarding biocompatibility, Fe_3_O_4_ and γ-Fe_2_O_3_ have been used in various formulations approved by the USA Food and Drug Administration (FDA) as a therapy for iron deficiency and as contrast agents for magnetic resonance imaging [14,25,26]. Magnetite (Fe_3_O_4_) also exhibits better electronic conductivity than other magnetic oxides in the same category due to its half-metallic nature with room temperature conductivity *σ* = 200 (Ω·cm)^−1^ [27,28]. In contrast, maghemite and hematite are semiconductors with bandgaps of approximately 2.0 eV, which are certainly less conductive than half-metallic materials [15,29]. Other spinel ferrites MFe_2_O_4_ are also mostly semiconductors [30,31]. In addition, Fe_3_O_4_ possesses the first-order transition of the Verwey transition (metal–insulator transition) at approximately 115–124 K, while this property is absent in maghemite [19,32]. Due to the Verwey transition properties, Fe_3_O_4_ is potentially very useful in various physical device applications. Other advantageous properties of Fe_3_O_4_ are its high electrochemical activity and high theoretical capacity, which are important for energy storage device applications [33]. In addition to these main properties, the natural abundance, inexpensiveness, and ecofriendliness are additional advantages that allow large-scale applications of Fe_3_O_4_-based NPs.

Numerous efforts to summarize progress in the synthesis, functionalization, nanoarchitectures, and applications of Fe_3_O_4_-based NPs have been reported. Several reviews have highlighted the use of iron oxide NPs (magnetite and maghemite) in biomedical applications [34–36]. The bioinspired synthesis and green biosynthesis of magnetite NPs have been summarized by Mirabello et al. [37] and Yew et al. [38]. Although the growth mechanism of Fe_3_O_4_ nanostructures and their applications were reviewed by Hou and coworkers in 2011 [17], numerous advances in the field have been achieved in the last 10 years. Other reviews have focused on special physical properties or effects, such as the Verwey transition [19] and exchange bias effects [16], which provide opportunities to integrate Fe_3_O_4_ NPs in electronic devices and physical instruments. Recently, Siregar et al. highlighted the use of Fe_3_O_4_ nanostructures in pollutant gas sensor systems [39], and Liu et al. reviewed synthetic methods and applications of Fe_3_O_4_ in multiple fields [18]. Despite the numerous available reviews, a comprehensive review focusing on the relationship of sizes and shapes (geometries) with the magnetic properties of Fe_3_O_4_ NPs, synthetic methods targeting each specific size and shape of Fe_3_O_4_ NPs, and preparations of appropriate nanoparticle systems for targeted applications is still needed [40–44]. We envision that the size-property and geometry-property relationships are very important factors contributing to the performance of Fe_3_O_4_ NPs in most applications. Therefore, this review will focus on the following problems:

- Synthetic methods to control the structures of Fe_3_O_4_ NPs with a focus on the sizes and geometries;
- Size- and geometry-to-magnetic property relationships of Fe_3_O_4_ NPs;
- Effects of size, geometries, and properties of NPs on target applications;
- Roles of functionalization and nanoarchitectures of Fe_3_O_4_ NPs in target applications.

**Figure 2.**
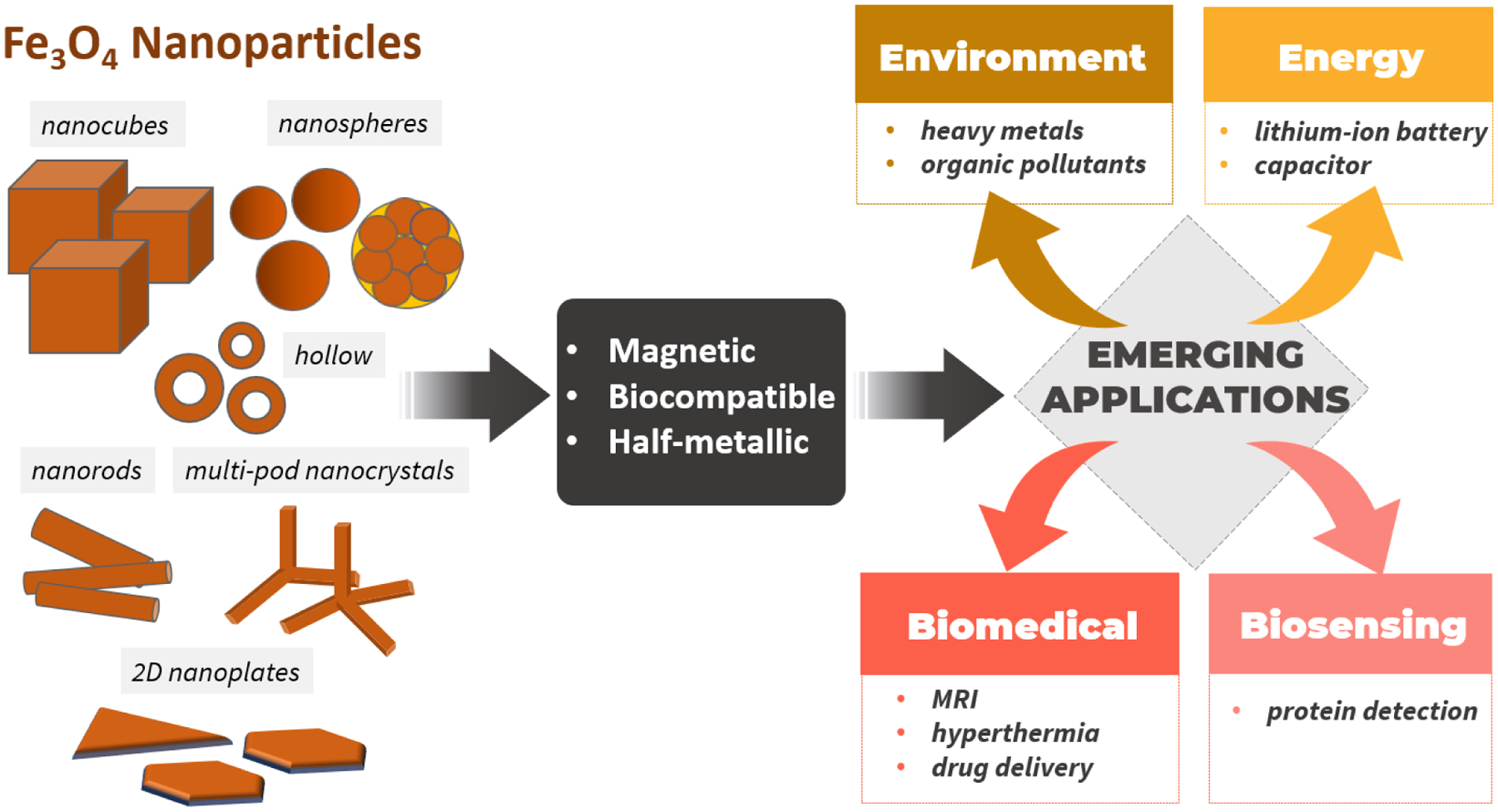
Fe_3_O_4_ NPs with various nanostructures and sizes used in emerging biomedical, biosensing, environmental, and energy applications.

We elucidate the solutions to these problems by first summarizing synthetic methods to obtain different nanostructures of Fe_3_O_4_ and their magnetic properties. In particular, the syntheses of various sizes of spherical, cubic, nanorod, 2D nanoplate (hexagonal and triangular shapes), hollow, and multipod nanocrystal Fe_3_O_4_ NPs are summarized together with their magnetic properties, including saturation magnetization and coercivity. In this section, synthetic strategies to tailor the size and morphology of NPs are mainly discussed. Next, we discuss the need to combine various characterization techniques to study Fe_3_O_4_ NPs. Then, we will highlight the use of Fe_3_O_4_-based NPs in emerging applications, such as biomedical applications (hyperthermia, MRI contrast agents, and drug delivery), biosensing, environmental applications for the removal of heavy metals and organic pollutants, and applications in energy storage devices. In this section, we will focus on the effects of the sizes, geometries, and thus magnetic properties of NPs, as well as the important roles of functionalization in enhancing the performance of Fe_3_O_4_ NPs in these applications. Figure 2 illustrates the scope of this review.

## 2. Structures, Synthesis, and Magnetic Properties of Fe_3_O_4_ Nanoparticles

**Figure 3.**
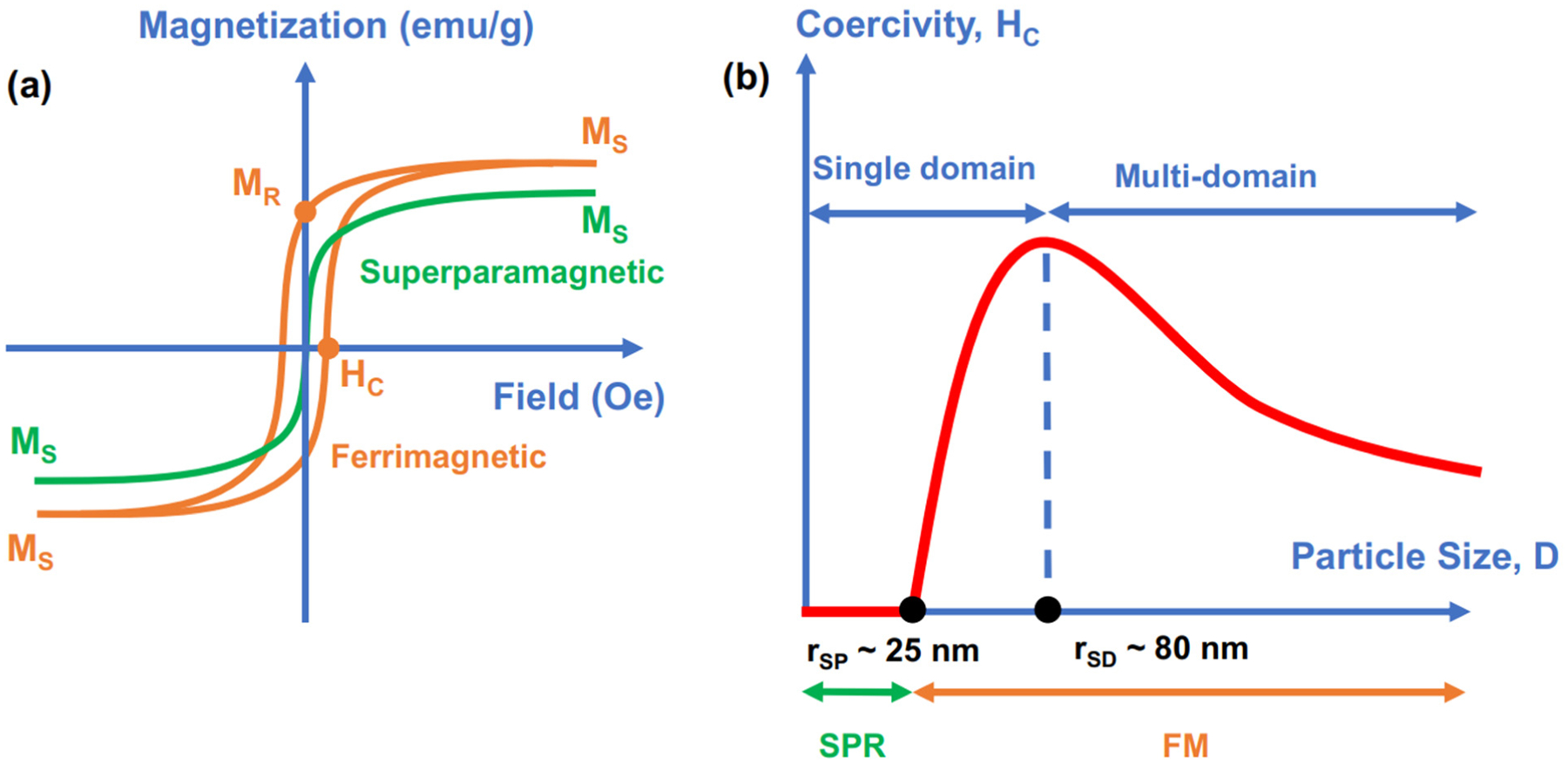
(**a**) Magnetization versus applied field (M–H) curves for superparamagnetic (SPM) (green color) and ferrimagnetic (FM) (orange) Fe_3_O_4_ nanoparticles and (**b**) relations between size, coercivity, and magnetic behavior.

Fe_3_O_4_ NPs exhibit either superparamagnetic (SPM) or ferrimagnetic (FM) behavior. Figure 3a shows examples of magnetization curves of the SPM curve (green) and FM hysteresis loop (orange color) of magnetic NPs represented by the magnetization value (emu/g) versus the applied magnetic field (Oe). In the presence of an external magnetic field, the magnetic material is magnetized and reaches a saturated value of magnetization called “saturation magnetization” (M_S_), which is the highest value of magnetization that the material can achieve. In addition to M_S_, the hysteresis curve of FM materials also exhibits coercivity (H_C_) and remnant magnetization (M_R_) values, which indicate how difficult the materials are to demagnetize and how much magnetization is retained in the absence of an applied magnetic field, respectively. For SPM NPs, H_C_ and M_R_ are equal to zero. Consequently, SPM materials do not exhibit magnetic properties without an applied magnetic field; however, they respond magnetically in the presence of an external magnetic field. SPM NPs have several advantages, such as preventing the agglomeration of NPs (caused by magnetic attraction) and a sensitive response to a remote-controlled magnetic field. In contrast, FM materials exhibit a certain magnetization value in the absence of an external magnetic field. Therefore, FM NPs always retain strong magnetic properties, which are potentially useful for applications that always require the existence of strong magnetic properties/signals.

At the nanoscale, Fe_3_O_4_ nanoparticles have different magnetic properties that are influenced by their structures, including size, morphology, crystallinity, and surface properties [2,42,45,46]. These parameters are strongly affected by the synthetic methods and chemicals used in their syntheses, such as iron precursors, surfactants, reducing agents, and solvents. Thus, the appropriate selection of a method to synthesize Fe_3_O_4_ NPs is very important to ensure the success of their applications. In the scope of this review, we focus on analyzing the magnetic features of Fe_3_O_4_ NPs at room temperature (RT) due to the special interest in their use in various technologies and applications. The superparamagnetic (SPM) and ferrimagnetic (FM) behaviors of Fe_3_O_4_ NPs depend on size, shape, crystallinity, and surface properties and are even affected by synthetic methods [46–50]. Figure 3b illustrates the relationship of the Fe_3_O_4_ NP size (diameter) with its magnetic behavior and coercivity. Two important size–magnetic property transitions of Fe_3_O_4_ NPs are superparamagnetic size (r_SP_) and single-domain size (r_SD_), which usually exist in NPs with diameters of ~25 nm and ~80 nm, respectively [45,51]. Superparamagnetic size (r_SP_) is the point at which the magnetic property of NPs transitions from the superparamagnetic to the ferromagnetic state, as NPs with a size larger than r_SP_ have a coercivity larger than 0 (H_C_ > 0). The features of the superparamagnetic behavior of NPs are coercivity and remnant magnetization equal to zero (H_C_ = 0 and M_R_ = 0) at temperatures above their blocking temperature (T_B_). In particular, NPs exhibit superparamagnetic behaviors at temperatures above T_B_ (T > T_B_) and ferromagnetic behavior at temperatures below T_B_ (T < T_B_). When the size of particles is adequately small (r < r_SP_), thermal energy overcomes anisotropy energy at the blocking temperature (T_B_), and nanoparticles become superparamagnetic. In our context, superparamagnetic NPs (at RT) should exhibit a blocking temperature much lower than room temperature. The single-domain size (r_SD_) is the size at which NPs spontaneously separate into multiple domains, causing a decrease in coercivity when the size of NPs increases. However, size is not a conclusive parameter that dictates the magnetic properties. Depending on the size, geometry, crystallinity, surface properties, and synthetic methods, the superparamagnetic–ferrimagnetic transition in Fe_3_O_4_ has been identified at approximately 20 nm [52], or even at ~30 nm [53]. Similar to the superparamagnetic–ferrimagnetic transition, the single-domain to multidomain transition occurs at different values and depends on the overall size, degree of crystallinity, and surface properties of NPs. Thus, the relationship between the size and magnetic properties of Fe_3_O_4_ NPs is not easy to predict due to various contributing factors.

In general, the magnetic properties of Fe_3_O_4_ NPs such as saturation magnetization (M_S_) and coercivity (H_C_) are strongly affected by four main factors including finite size effects, surface effects, magnetic anisotropy, and the degree of crystallinity [2,5,42,45,46]. These factors are correlated to each other and strongly influenced by the size and geometry of the NPs. The finite size effects are typically related to special behaviors of a substance in a finite nanoscale size, such as quantum confinement of electrons [2]. In nano-magnetism, the single-domain limit and the superparamagnetic limit (presented in Figure 3) are the most studied finite size effects, which typically dictate the magnetic behavior of particles for ferrimagnetic and superparamagnetic responses, respectively [2]. While the superparamagnetic limit has been discussed in the previous paragraph, the single-domain limit is driven by balancing magnetostatic energy and domain wall energy, which induces the formation of magnetic domains [2]. Thus, the finite size effect of the single-domain limit regulates the change in ferrimagnetic properties, such as the change in coercivity in ferrimagnetic NPs when the size of the NPs increases. As illustrated in Figure 3b, increasing the size of NPs boosts H_C_ until the specific size limit called the “single-domain limitation” is reached, where the separation of multiple domains begins and induces the decrease in H_C_.

The surface effect has been used to rationalize the decrease in M_S_ of smaller Fe_3_O_4_ NPs due to a surface-disordered spin layer [45]. For small NPs, the ratio of surface atoms to bulk atoms increases, giving rise to more significant contributions of surface spins to magnetization. The detrimental role of the surface effect on magnetization can be rationalized by various contributions, such as canted spins, magnetically dead layers, and spin glass-like behavior of surface spins [2]. As a consequence, the surfactants coated on the NPs also alter the magnetic properties of NPs, which can either positively or negatively affect the surface spin. Thus, the size of NPs can help to predict the change in M_S_; however, it is not the conclusive factor. In addition, as different geometries of NPs have different ratios of surface atoms, Fe_3_O_4_ NPs with comparable sizes but having different shapes can exhibit different magnetic properties. Magnetic anisotropy describes the directional dependence of the magnetic moment in materials. Magnetic anisotropy is a crucial factor contributing to the effect of the size and geometry on magnetic properties. In this context, magnetic anisotropy is related to magnetocrystalline anisotropy and shape anisotropy, which correspond to the preferential magnetization direction of the crystals and the departure from sphericity of particle shapes, respectively [2]. The magnetic anisotropy of nanoparticles can be higher than the value obtained from the crystalline and shape anisotropy due to enhanced surface anisotropy [2]. Furthermore, shape anisotropy is also a vital factor to determine the strength of the magnetic properties of magnetic NPs [2,5]. Finally, highly crystalline structures can significantly enhance the magnetic properties of NPs, even NPs with comparable sizes and shapes [46,47]. Enhanced crystallinity was also proposed as the reason for increasing the magnetic properties of cubic versus spherical Fe_3_O_4_ NPs [42]. These contributing factors, driven by the size and geometries of Fe_3_O_4_ NPs, underpin the fundamental phenomena that rationalize or predict the magnetic properties when the size and geometries of Fe_3_O_4_ NPs are varied.

Due to the strong effects of the geometry and size on the properties of NPs, we will summarize the advances in the synthesis of Fe_3_O_4_ NPs with different geometries and sizes. For a particular geometry, we will cover the synthesis routes for different size ranges and compare the important properties (e.g., saturated magnetization M_S_ and coercivity H_C_) of these NPs. We focus on three geometries, spherical, cubic, and rod, and highlight recent advances in the synthesis of other sophisticated geometries, such as 2D hexagonal/triangular shapes, multiarmed structures, octahedrons, and hollow structures. We focus on methods with high efficiency, good control of geometry uniformity, narrow size distributions, and the ability to tune the size of particles.

### 2.1. Fe_3_O_4_ Spherical Nanoparticles (SNPs)

In this section, Fe_3_O_4_ spherical nanoparticles (SNPs) will be categorized into two size ranges based on their structures, crystallinity, and magnetic behaviors. The first type is Fe_3_O_4_ SNPs with a size smaller than 25 nm and a single-crystalline structure that probably exhibits superparamagnetic behavior at room temperature. This type of SNP is widely applied in hyperthermia, as a contrast agent in magnetic resonance imaging, and in drug delivery. Table 1 summarizes the available synthetic methods and the strategy for controlling the size to obtain single-crystalline Fe_3_O_4_ SNPs with a size less than 25 nm, as well as the magnetic properties of saturation magnetization at room temperature for comparisons of these SNPs. The second type of SNP is usually polycrystalline NPs with diameters generally larger than 25 nm to even hundreds of nanometers. The structure of these Fe_3_O_4_ SNPs is often characterized by the stacking or agglomeration of small primary nanocrystals to form larger SNPs as the secondary structure. In this size range, the magnetic properties of SNPs at room temperature are either ferrimagnetic or superparamagnetic, depending on the crystallite size, the overall size of NPs, and the interaction of nanosized subunits. Together with summarizing the available synthetic methods of Fe_3_O_4_ SNPs with sizes ranging from 25 nm to a few hundred nanometers, Table 2 also provides detailed information on the particle size, crystallite size, and magnetic properties, including M_S_ and H_C_, at room temperature.

**Figure 4.**
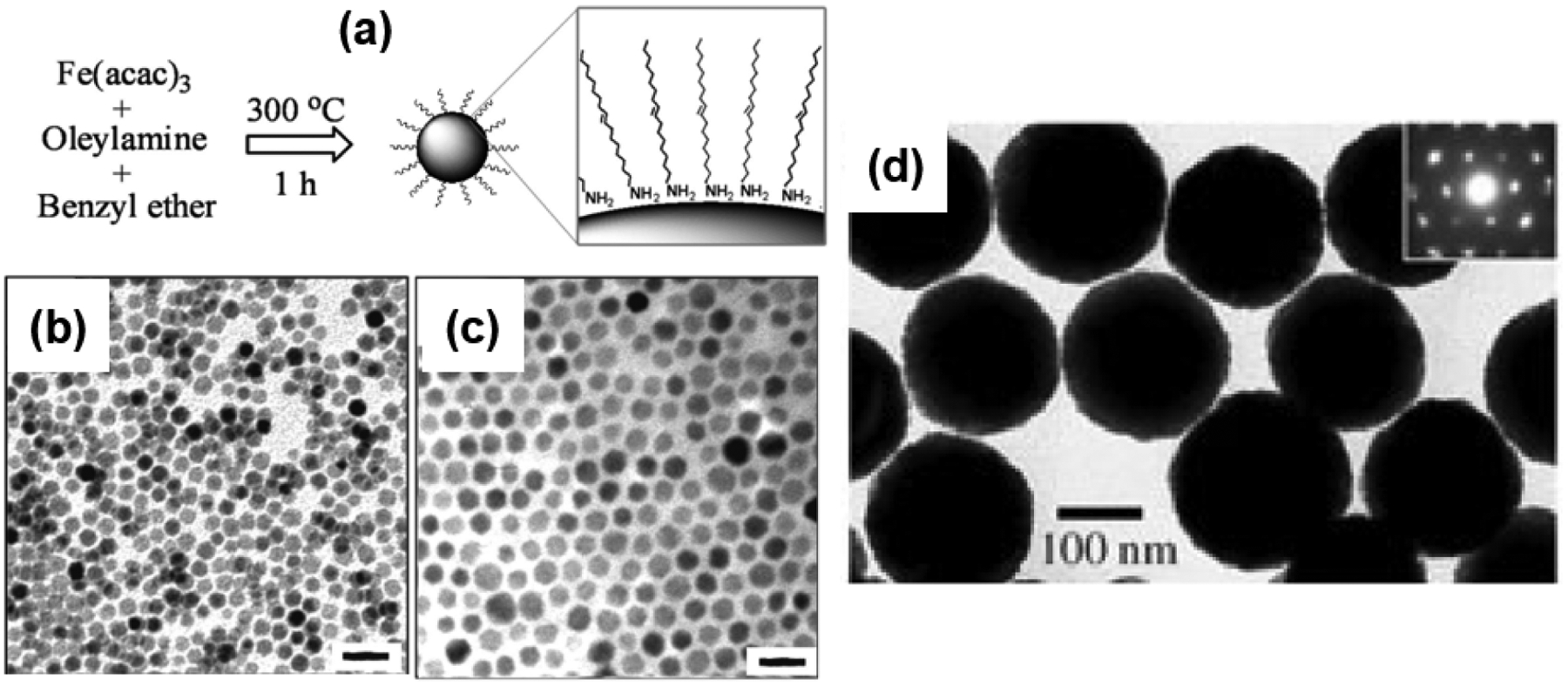
(**a**) Schematic synthesis of Fe_3_O_4_ NPs with sizes of 7, 8, 9, and 10 nm. Selected TEM images of Fe_3_O_4_ NPs with sizes of (**b**) 7 ± 0.5 nm and (**c**) 10 ± 0.8 nm. Scale bar 20 nm. Reproduced with permission from ref. [57]. Copyright 2009 American Chemical Society. (**d**) TEM image of spherical Fe_3_O_4_ NPs with a size of 200 nm. Reproduced with permission from ref. [48]. Copyright 2005 John Wiley and Sons.

Thermal decomposition of iron complexes at high temperature in high-boiling point organic solvents has proven to be an effective method to generate Fe_3_O_4_ nanospheres with sizes ranging from 4 to 30 nm [54–58]. Sun et al. reported the thermal decomposition of Fe(acac)_3_ in phenyl ether (boiling point: 260 °C) at 265 °C to synthesize monodisperse 4 nm Fe_3_O_4_ SNPs [54]. 1,2-Hexadecanediol was used as the reducing agent, and a mixture of oleic acid (OA) and oleylamine (OAm) was utilized as the surfactant. The as-synthesized 4 nm NPs were then used as seeds for seed-mediated growth to obtain 8, 16, and 20 nm SNPs with the assistance of stearyl alcohol. Larger seeds with an average diameter of 6 nm were prepared by refluxing at a higher temperature of 300 °C in the higher-boiling point benzyl ether solvent (boiling point: 296 °C) [55]. In this method, 1,2-hydrocarbon diols served as excellent reducing agents for the synthesis of high-quality Fe_3_O_4_ SNPs with good yields for use as seed NPs. Interestingly, the seed-mediated growth processes normally require long-chain mono-alcohols to grow larger particles rather than diol derivatives. Later, Xu et al. [57] reported a more convenient one-pot method to synthesize Fe_3_O_4_ nanospheres with sizes of 7, 8, 9, and 10 nm by simply controlling the ratio of OAm and benzyl ether, as shown in Figure 4a. Here, oleylamine served as a multifunctional reagent: a strong reductive agent and an effective capping agent [57,65]. The obtained NPs exhibited a homogeneous size and uniform spherical morphology, as verified using transmission electron microscopy (TEM). Selected TEM images of Fe_3_O_4_ SNPs with sizes of 7 and 10 nm are presented in Figure 4b,c, respectively. The heating procedure is very important in the thermal decomposition of Fe(acac)_3_, which usually requires maintenance at 200 °C for nucleation prior to refluxing or a quick ramping rate of 20 °C/min. Therefore, precisely controlling the temperature of the reaction and a powerful heating process represent a technical challenge for large-scale production. Later, Lee and coworkers successfully lowered the reflux temperature to 200 °C using alkaline metal reagents to assist with the reduction of iron precursors [58]. In the presence of Mg(acetate)_2_, monodisperse Fe_3_O_4_ SNPs with sizes of 8, 11, 15, and 18 nm were prepared. Park et al. reported an ultralarge-scale synthesis with 40 g of products obtained per single reaction using the inexpensive and environmentally friendly starting material iron(III) chloride [56]. Nanospheres with sizes of 5, 9, 12, 16, and 22 nm were prepared with a uniform size (size variation < 4.1%) and homogeneous morphology. Iron(III) oleate complexes were first prepared from iron(III) chlorides and sodium oleate before refluxing with oleic acid at 320 °C in different high-boiling point organic solvents to obtain SNPs of different sizes. In addition to the advantages of highly uniform size and morphology, NPs prepared using thermal decomposition usually have hydrophobic surfaces that require surface modifications for applications requiring water-soluble nanosubstances, such as biomedical applications or catalysis in aqueous media. Li and coworkers approached this problem by applying surfactant-free thermal decomposition of the inexpensive precursor FeCl_3_·6H_2_O in a strongly polar 2-pyrrolidone solvent [60]. This recipe allowed obtaining SNPs in a wider range of sizes from 4 to 60 nm by simply controlling the reaction time. Thus, thermal decomposition is a useful synthesis method producing high-quality Fe_3_O_4_ SNPs with good uniformity in size and morphology.

In addition to thermal decomposition, solvothermal methods are also used to synthesize spherical superparamagnetic NPs in this size range below 25 nm at lower temperatures than thermal decomposition methods. Caruntu et al. prepared Fe_3_O_4_ SNPs with sizes ranging from 6.6 to 17.8 nm through the solvothermal mixing of FeCl_2_ and FeCl_3_ precursors in solvent mixtures using different ratios of diethylene glycol and N-methyl diethanolamine [63]. Fe(acac)_3_ has also been used as a precursor in the solvothermal synthesis of 8 and 11 nm SNPs by employing different surfactants [59]. In addition, direct preparation of SNPs with hydrophilic surfaces has also been developed [18,66]. However, the size distribution and uniformity of the spherical geometry are not as good as SNPs prepared using thermal decomposition at high temperatures. For example, Ge and coworkers synthesized 15 to 31 nm NPs by oxidizing FeCl_2_·4H_2_O in a basic aqueous solution [61]. These NPs exhibit the gradual transition from ferromagnetic to superparamagnetic when decreasing in size. Kim and colleagues developed a facile route for the large-scale sonochemical synthesis of 11 nm Fe_3_O_4_ SNPs with M_S_ = 80 emu/g using inexpensive and nontoxic reactants, such as FeCl_2_, FeCl_3_, and H_2_O [64]. Thus, among the various synthesis methods, thermal decomposition is the most effective for the synthesis of Fe_3_O_4_ nanospheres with a size less than 25 nm. The size and uniformity of NPs can be effectively controlled in this method by modifying the amounts of surfactants, heating protocol, reducing agents, or solvent compositions. The main drawback of this method is the technical requirements for maintaining high temperatures and fast heating rates.

Spherical particles in the size range from 25 to even a few hundred nanometers have been synthesized successfully using various recipes and solvothermal methods [42,43,48]. In this size range, spherical Fe_3_O_4_ NPs exhibit diverse magnetic behaviors, either superparamagnetic or ferrimagnetic (Table 2). In contrast to single-crystalline SNPs with sizes below 25 nm, magnetite particles in the larger size range (diameter > 25 nm) are usually polycrystalline NPs. These NPs are composed of numerous primary nanocrystals aggregated to form secondary structure NPs. Consequently, the magnetic properties of these nanospheres depend on the size of the nanosized subunits, the overall size of the nanospheres, interactions of the primary crystals, surface properties, and morphology. These NPs are either ferrimagnetic or superparamagnetic, depending on both their overall size and the size of the subunits. These types of nanoparticles are also called “colloidal nanocrystal clusters” (CNCs) [53] or colloidal superparticles (SPs) [67,68]. For example, Zhuang and coworkers prepared iron oxide NPs with a size of 5.8 nm through the thermal decomposition of iron(III) oleate [56] and then used nanoparticle micelle formation to form larger supercrystalline colloidal SPs with sizes of 120, 190, and 560 nm via solvophobic interactions [67,68]. These colloidal SPs have superparamagnetic properties at room temperature along with excellent stability in polar solvents. Deng et al. reported single-crystalline, monodisperse ferrite NPs with tunable sizes ranging from 200 to 800 nm using a solvothermal reduction method [48]. A TEM image of the obtained mean 200 nm spherical Fe_3_O_4_ NPs is shown in Figure 4d. Later, this method was widely applied to synthesize SNPs for various applications, including protein detection [43] and drug delivery [69]. In the presence of a polyethylene glycol (PEG) surfactant, FeCl_3_·6H_2_O was reduced in ethylene glycol at 200 °C assisted by sodium acetate, an electrostatic stabilizer and a reductive-mediated agent. The size of NPs is simply controlled by the reaction time in the range of 8 to 72 h. Lee and colleagues modified this recipe by adding different surfactants, polyvinylpyrrolidone, refluxing at 180 °C, and manipulating the reaction time from 4 to 24 h to generate NPs with diameters ranging from 100 to 275 nm [42]. Separately, Yin and coworkers successfully synthesized highly water-dispersible Fe_3_O_4_ SNPs with sizes from 30 to 180 nm by controlling the hydrolysis of FeCl_3_ during high-temperature reduction in diethylene glycol [53]. In this recipe, poly(acrylic acid) (PAA) was used as the surfactant and dictated the release of water for hydrolysis upon the addition of NaOH. Thus, the size of NPs can be tuned by adding different amounts of NaOH stock solution in DEG. The packing of small primary crystals with a size of approximately 10 nm to form colloidal nanocrystal clusters (mean size 30–180 nm) has many advantages, such as maintaining superparamagnetic behavior and water-dispersible properties, as well as enhancing the overall magnetization of the nanoparticles. In addition to synthesizing different sizes of secondary structure SNPs with similar crystalline subunits, the preparation of NPs of similar sizes with a tunable degree of crystallinity was reported by Xuan and coworkers. They developed a method to prepare secondary structures of CNCs with a diameter of 280 nm that have tunable crystallite sizes ranging from 5.9 to 21.5 nm [46]. By employing different ratios of sodium acetate and sodium acrylate, grain sizes were controlled without changing the overall size of NPs. Consequently, similar sizes of NPs with different magnetization values were obtained. Moreover, a novel strategy to tune the size of secondary structural Fe_3_O_4_ SNPs from 6 to 170 nm was introduced that used different solvent mixture compositions of EG and DEG. Due to its bulky molecules, DEG slowed the aggregation of primary crystals and created more seeds for growing NPs, causing smaller NPs to form [43,46,47]. Utilizing this binary solvent system, the size of spherical NPs was even manipulated in a wider range from 20 to 300 nm [47]. These NPs have either superparamagnetic or ferrimagnetic properties that are dictated by varying the water concentration in the synthesis reaction. Liu et al. studied the effect of the water volume fraction on the crystallite sizes and sizes of NPs in solvothermal synthesis using ethylene glycol as the solvent [70]. They found that the overall size of particles exhibited an extremely wide range (82 to 1118 nm) when the volume percentage of added water increased (from 5.5% to 20.5%). The crystallite size of NPs changes with an increasing water volume ratio and follows an inverse U-shaped curve, with the peak located at a 14.5% water volume ratio. In another study, Chen et al. used a solvent mixture composed of a 1/3 ratio of EG/DEG to obtain 100 nm Fe_3_O_4_ NPs and only an EG solvent with a higher concentration of iron precursors to prepare magnetite NPs with sizes of 440 and 720 nm [43]. These NPs were prepared in a pressure vessel at 188 °C under continuous vigorous agitation. Based on these results, which are summarized in Table 2, the solvothermal reduction of the FeCl_3_ precursor and the controlled hydrolysis of iron cations are useful methods to prepare Fe_3_O_4_ in a wide range of sizes, from a few tenths to a few hundred nm. These Fe_3_O_4_ NPs have tunable degrees of crystallinity and exhibit different magnetic properties, ranging from superparamagnetic to ferromagnetic. With featured properties and size characteristics, these spherical Fe_3_O_4_ nanoparticles can be used in multiple applications, such as sensing, biomedical applications, environmental remedies, and catalysis.

### 2.2. Fe_3_O_4_ Cubic Nanoparticles (CNPs)

Fe_3_O_4_ cubic nanoparticles (CNPs) exhibit either superparamagnetic or ferromagnetic behaviors at room temperature, depending on the size of the NPs and the synthetic methods. However, the preparation of cubic magnetite nanocrystals seems to be more challenging than the preparation of spherical NPs. With the nature of a cubic crystal structure, Fe_3_O_4_ tends to undergo isotropic growth that easily results in spherical particles. Several studies demonstrated that CNPs possess better crystallinity and stronger magnetic properties than their spherical counterparts with equivalent sizes [41,42,71]. Due to the challenge of maintaining dominant growth along <111> surfaces, only a limited number of synthetic methods are available for the preparation of CNPs with sizes ranging from approximately 10 to 180 nm [49,50,72]. The available synthetic methods, size control factors, and magnetic properties of the corresponding Fe_3_O_4_ CNPs are summarized in Table 3.

Kovalenko and coworkers performed the thermal decomposition of iron(III) oleate in the presence of a sodium oleate surfactant to prepare superparamagnetic cubic Fe_3_O_4_ with edge dimensions of 9.3, 13.4, 15.5, and 22.1 nm [73]. Similarly, Yang et al. utilized Fe(acac)_3_ as a precursor together with 1,2-hexadecandiol, oleic acid, and oleylamine at a high temperature (290 °C) [49]. By increasing the heating rate from 5 to 35 °C/min and shortening the reaction time from 3 h to 20 min, monodisperse nanocubes with controllable sizes ranging from 6.5 to 30 nm were prepared. Hyeon and colleagues reported a simple route using only two reagents, Fe(acac)_3_ (precursors) and oleic acid (surfactant) [50]. This mixture with a precise 1:2 molar ratio of Fe(acac)_3_ and oleic acid was degassed and then refluxed at 290 °C in benzyl ether with a fast ramping rate of 20 °C/min. By controlling the concentration of reagents and reaction time, Fe_3_O_4_ nanocubes with edge lengths of 79 and 160 nm were obtained. Surprisingly, 1.6 g of magnetite nanocubes with an edge length of 49 nm can be synthesized by scaling up the recipe for the 79 nm nanocubes 10 times. Biphenylcarboxylic acid can be introduced in combination with oleic acid to direct the growth of 22 nm Fe_3_O_4_ nanocubes as a method to reduce the nanocube size. Lee and colleagues applied this synthetic approach to prepare Fe_3_O_4_ nanocubes with sizes ranging from 78 to 130 nm and explored their biosensing potential [42]. The authors also observed significantly higher magnetic properties of the Fe_3_O_4_ nanocubes compared to the nanospheres with a similar volume or comparable diameter/body diagonal dimensions. Using a different fatty acid in the thermal decomposition of Fe(acac)_3_, Guardia et al. remarkably controlled the sizes of magnetite cubic particles in a broad range from 13 to 180 nm by adjusting the heating rate [72]. Specifically, the mixture of decanoic acid and iron(III) acetylacetonate with a 4-to-1 molar ratio was first heated to 60 °C for degassing, subsequently ramped to 200 °C, and finally refluxed at 290 °C with a slow ramping rate from 0.8 to 5.2 °C/min. Pellegrino and colleagues applied this recipe with modifications to synthesize iron oxide nanocubes with sizes ranging from 12 to 38 nm and investigated their performance in cancer hyperthermia treatments [52]. In addition, solvent mixtures of squalene and benzyl ether were used to overcome the unstable temperature problem of a pure benzyl ether solvent during the reaction [44]. This binary solvent system allowed more precise control of the reaction temperature and improved reproducibility.

**Figure 5.**
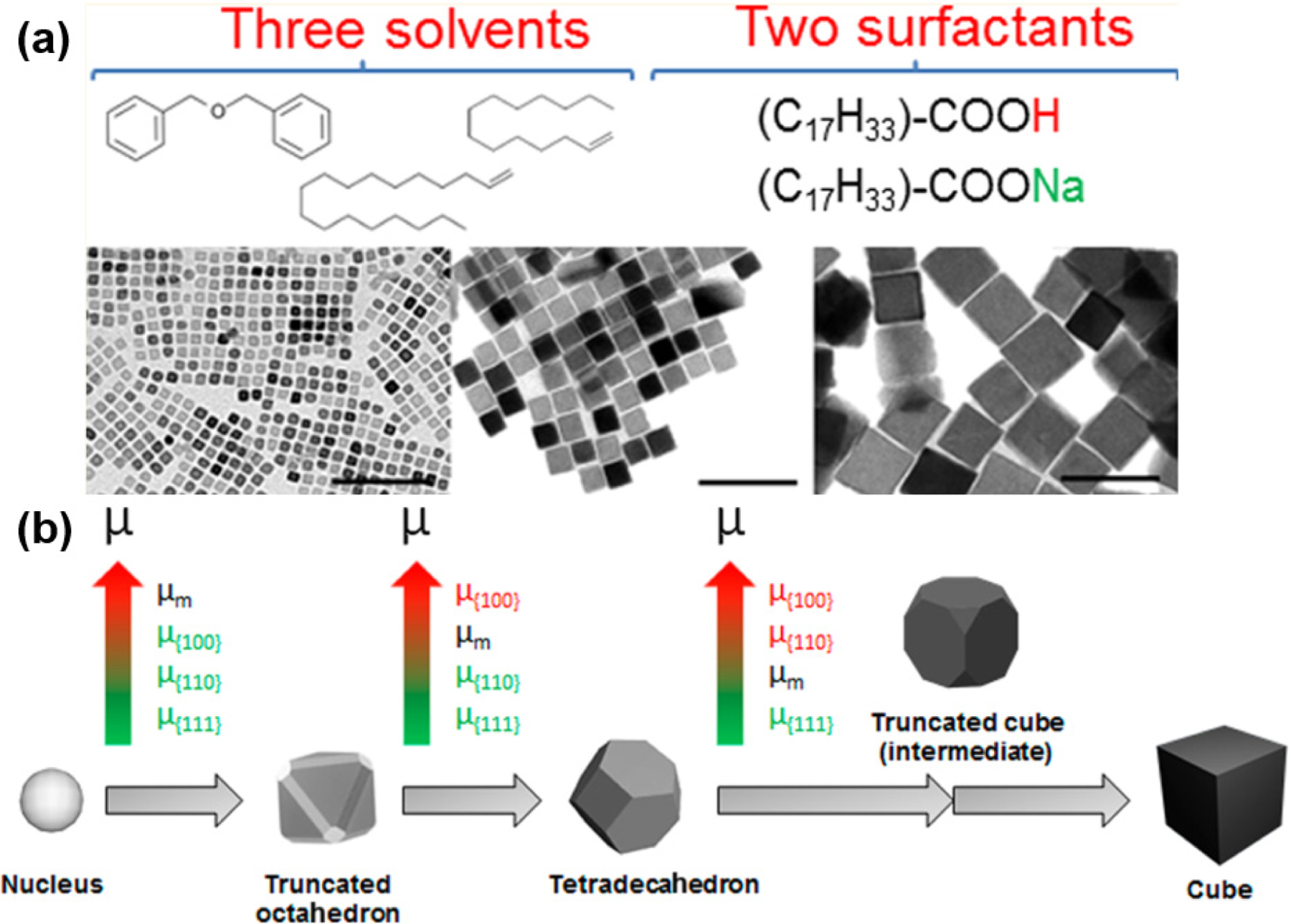
(**a**) Schematics showing the synthesis of Fe_3_O_4_ nanocubes with edge lengths in the 9–80 nm range and (**b**) the growth mechanism of Fe_3_O_4_ nanocubes. Reproduced with permission from ref. [74]. Copyright 2019 American Chemical Society.

Lee and collaborators reported the synthesis of magnetite nanocubes with sizes of 22, 36, and 57 nm at a lower refluxing temperature (200 °C) using an alkaline metal acetate to assist with the reduction process [58]. The amount of sodium oleate surfactant was found to play an important role in controlling the size of nanocubes. Recently, significant progress in understanding the mechanism and size-controllable synthesis was reported by Muro-Cruces, Roca, and coworkers. They used a binary surfactant system containing oleic acid and sodium oleate in mixtures of three solvents (1-octadecene, dibenzyl ether, and 1-tetradecene) to obtain nanocubes with uniform sizes ranging from 9 to 80 nm [74]. Benzyl ether was again identified as a good solvent for the dispersion of Fe(acac)_3_ precursors; however, it is not stable at high temperatures, producing volatile products such as benzyl aldehyde or benzyl benzoate [44,76]. Thus, the combination of 1-octadecene, dibenzyl ether, and 1-tetradecene was utilized to compromise between the good dispersion of precursors and maintain a stable temperature during synthesis. Figure 5a presents the synthetic strategy using a combination of three solvents and two surfactants to synthesize Fe_3_O_4_ nanocubes in a 9–80 nm size range. The growth mechanism is proposed in Figure 5b, starting from the nucleus to the truncated octahedron, then the tetradecahedron, and finally the nanocubes. In this figure, the chemical potentials of crystal facets are presented from low to high, as indicated by a green-to-red color scale. In addition to thermal decomposition, magnetite nanocubes have also been synthesized using sonochemistry [75,77], precipitation [78], and solvothermal green synthesis [71]. However, thermal decomposition at high temperatures assisted by fatty acid ligands is the most efficient method that provides uniform cubic geometries, a narrow size distribution, and tunable sizes in a wide range from approximately 10 to 180 nm.

### 2.3. Other Geometries

The isotropic spherical geometry and anisotropic cubic geometry are two morphologies of Fe_3_O_4_ nanoparticles that have been extensively studied in synthesis and applications. Moreover, various anisotropic or special geometries of Fe_3_O_4_ nanoparticles have been synthesized, such as the 1D structures of nanorods and nanotubes and the 2D structures of nanoprisms, hexagonal nanoplates, multiarmed nanostars, tetrapods, and hollow nanoparticles [17]. Detailed descriptions of the sizes, morphologies, and related magnetic properties of these special geometries of Fe_3_O_4_ NPs are presented in Table 4.

**Figure 6.**
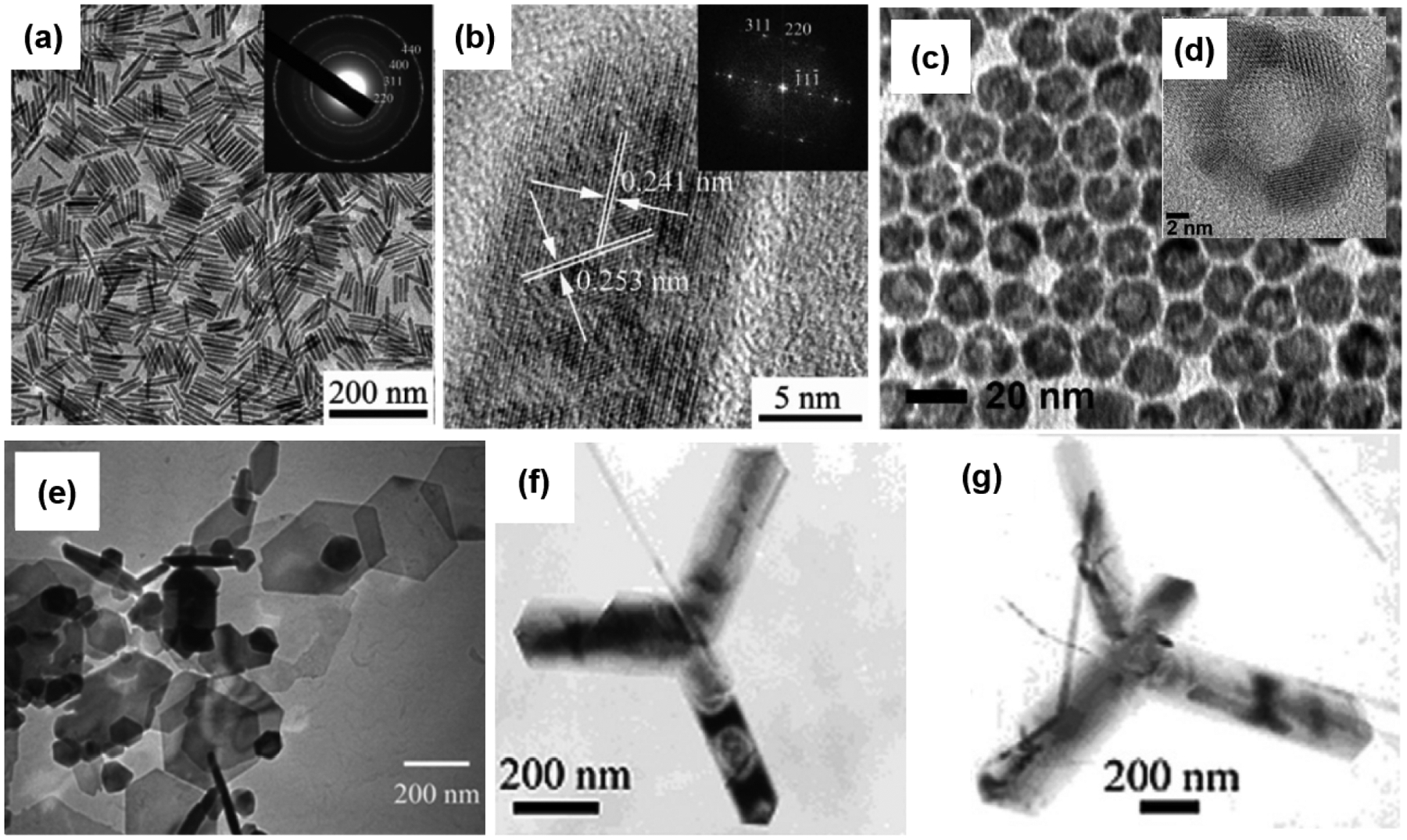
(**a**,**b**) TEM and HR-TEM images of Fe_3_O_4_ nanorods. Reproduced with permission from ref. [79]. Copyright 2012 American Chemical Society. (**c**,**d**) TEM and HR-TEM images of porous hollow NPs. Reproduced with permission from ref. [87]. Copyright 2009 American Chemical Society. (**e**) TEM image of 2D hexagonal nanoplates. Reproduced with permission from ref. [85]. Copyright 2010 American Chemical Society. (**f**,**g**) TEM images of Fe_3_O_4_ tripods and tetrapods. Reproduced with permission from ref. [86]. Copyright 2009 Elsevier.

For simple one-dimensional morphologies and highly anisotropic shapes, single-crystalline Fe_3_O_4_ nanorods were synthesized through the solvothermal synthesis of an iron pentacarbonyl precursor in octanol using hexadecylamine and oleic acid coordinating agents [79]. By adjusting the amount of hexadecylamine and reaction time, the sizes of nanorods or aspect ratios (length-to-diameter ratio) can be controlled. Two different sizes, length × diameter (L × D) of 65 × 6.5 nm and 140 × 12 nm, were observed with TEM, and HR-TEM images of the obtained Fe_3_O_4_ nanorods are shown in Figure 6a,b [79]. Separately, Das and coworkers synthesized Fe_3_O_4_ nanorods with different aspect ratios ranging from 5.6 to 11. They also reported the synthesis of Fe_3_O_4_ nanorods with comparable aspect ratios but different lengths and diameters [40]. A mechanistic study of the reaction revealed that Fe_3_O_4_ nanorods are formed by heteronucleation on intermediate FeO nanocubes [79]. In this reaction, Fe(CO)_5_ is first decomposed under solvothermal conditions to generate metallic iron. Then, metallic iron is oxidized by dissolved oxygen or other oxidizing species in the solution to form FeO nanocubes. At the same time, Fe(CO)_5_ reacts with oleic acid to form iron oleate simultaneously to the condensation of hexadecylamine and oleic acid to release water molecules for the hydrolysis of iron oleate. Combined with the dissolution of FeO, the decomposition of Fe(CO)_5_ and the hydrolysis of iron oleate provide the source for growing Fe_3_O_4_ nanorods. Applying this method, Chandra et al. fabricated epitaxial magnetite nanorods on a SrTiO_3_ substrate and observed enhanced room temperature magnetic anisotropy [90]. Nanorods of Fe_3_O_4_ have also been synthesized using a two-step process starting with the preparation of β-FeOOH nanorods before refluxing in oleylamine to form the Fe_3_O_4_ phase [80]. This method allows the preparation of nanorods with a wide range of controllable lengths from 35 to 180 nm, and the diameter can be tuned from 5.5 to 24 nm. Larger dextran-coated nanorods with average dimensions of L × D 310 × 135 nm were synthesized using a precipitation method [81]. Surprisingly, these large nanorods are retained in the superparamagnetic regime and have potential applications as spin–spin relaxation contrast agents and in monitoring peroxidase activity.

Two-dimensional nanoplates of Fe_3_O_4_ with hexagonal or triangular geometries have also been synthesized using various different methods. Single-crystalline Fe_3_O_4_ nanoplates, consisting of triangular and hexagonal shapes, were synthesized with a facile template-free solvothermal method [82]. These nanoplates have an average thickness of ~7 nm, a mean width of ~120 nm for hexagonal shapes, and a ~90 nm side length for triangular shapes. Interestingly, due to the shape anisotropy of nanoplates, the nanoplates exhibit an extremely high coercivity of 117.72 Oe, which is higher than the coercive fields observed for spheres, polyhedral, solid, or hollow spheres (usually less than 110 Oe). Li et al. also applied a simple hydrothermal method using 1,3-propanediamine to prepare single-crystalline Fe_3_O_4_ triangular nanoprisms with an average edge length of 113 nm and a mean thickness of ~25 nm [83]. Triangular nanoprisms have also been synthesized by the decomposition of Fe(acac)_3_ in toluene using oleylamine as the surfactant and reducing agent [84]. In addition to wet syntheses, the supercritical fluid technique was also applied to synthesize Fe_3_O_4_ hexagonal nanoplatelets. This method utilized ferrocene as a precursor and supercritical carbon dioxide (sc-CO_2_) as the solvent and oxygen sources [85]. As shown in Figure 6e, the obtained hexagonal plates are uniform with a mean diameter of 200 nm and thickness ranging from 20 to 30 nm.

In addition to anisotropic shapes such as 1D nanorods and 2D nanoplates, a special geometry of multiarmed nanocrystals containing Y-shaped bipods, tripods, and tetrapods was successfully synthesized with a simple route using a mild hydrothermal protocol. This special geometry of multiarmed nanostructures exhibited an extremely high saturation magnetization of 106.6 emu/g, which is even higher than the maximum M_S_ value of 92 emu/g for bulk magnetite [86]. TEM images of the tripod and tetrapod Fe_3_O_4_ nanocrystals are shown in Figure 6f,g, respectively. Moreover, a special version of the isotropic morphology of SNPs with hollow nanostructures was also synthesized [87,88]. Because they possess porous shells, these Fe_3_O_4_ hollow nanostructures are potentially useful for drug delivery applications. Separately, 19 nm porous hollow Fe_3_O_4_ nanoparticles were prepared through the controlled oxidation of Fe NPs, followed by an acid etching step [87]. The morphology and structure of these porous hollow NPs are presented in Figure 6c,d. Larger hollow Fe_3_O_4_ NPs with a mean diameter of 120 or 295 nm were also prepared using a direct solvothermal method or a combination of a solvothermal route and etching, respectively [88,89].

**Figure 7.**
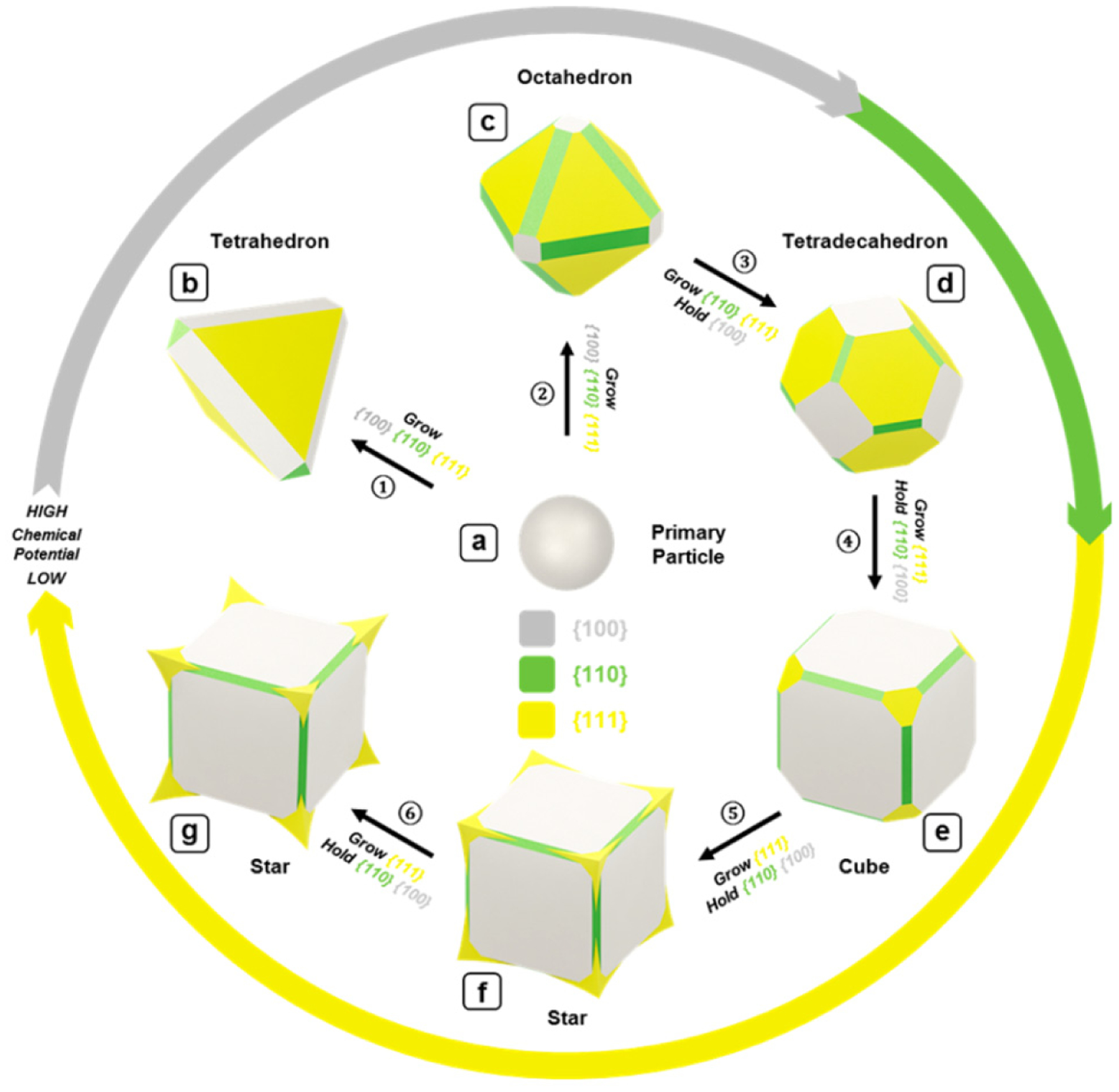
Schematic illustration of the proposed growth model for MNCs. Reproduced with permission from ref. [76]. Copyright 2017 American Chemical Society.

An atlas of synthetic strategies for synthesizing various geometries of magnetite nanoparticles, such as tetrahedrons, octahedrons, tetradecahedrons, cubes, and stars, was developed by Swihart and coworkers [76]. The authors proposed the growing mechanism of Fe_3_O_4_ nanocubes during the synthesis process and provided controllable growth procedures to obtain different geometries, such as tetrahedrons, octahedrons, and star shapes (cubic with sharply extruded corners), as shown in Figure 7.

Significant efforts have been made in the last 20 years to synthesize Fe_3_O_4_ nanoparticles with highly pure compositions, controllable sizes in a wide range, good uniform geometry, and high degrees of crystallinity. Furthermore, the in-depth mechanism of some synthetic protocols has been explained, which provides a better understanding of nanochemistry during synthesis processes and allows the tailoring of nanoparticle properties. In terms of synthesis, spherical Fe_3_O_4_ nanoparticles are relatively easily prepared and available in a wide range of sizes for use in various applications. Compared to spherical NPs, cubic Fe_3_O_4_ NPs show enhanced magnetic properties; however, the uniform growth of cubic crystals is more difficult to control. Although Fe_3_O_4_ nanorods possess large surface contact areas, they exhibit moderate magnetic properties. Some special geometries have excellent magnetic properties, such as 2D nanoplates with high coercivity and multiarmed nanocrystals with high saturation magnetization. Thus, this review provides a brief summary of the preparation of Fe_3_O_4_ NPs with specific sizes, geometries, and magnetic properties, which may be helpful for the preselection of synthetic routes for any target application.

## 3. Techniques for Characterizing Fe_3_O_4_ NPs

A combination of different methods is usually required to identify the composition and purity and evaluate the properties of Fe_3_O_4_ NPs. The most important aspect is to distinguish Fe_3_O_4_ from the maghemite phase and identify the compositional uniformity. Because the oxidation of Fe^2+^ to Fe^3+^ is a thermodynamically favored process, the coexistence of magnetite and maghemite phases is usually observed in Fe_3_O_4_ nanoparticles [56,91]. The presence of the maghemite phase in the magnetite nanoparticles may lead to a decrease in saturation magnetization. X-ray diffraction (XRD) is generally unable to distinguish between magnetite and maghemite phases due to similar patterns originating from the same cubic spinel structures [91]. However, two features that can be deduced from XRD are matching lattice parameters, and d-spacings have been used to further confirm the existence of the dominant magnetite phase [50,54,57]. For example, a slight difference in the standard lattice parameters of magnetite (8.396 Å) and maghemite (8.346 Å) has been identified [57]. X-ray photoelectron spectroscopy (XPS) is an effective method to prove the existence of the magnetite phase because of the coexistence of Fe^2+^ and Fe^3+^ cations [92]. Infrared spectroscopy [66,93] and Raman spectroscopy [94,95] are also widely used to assign and identify magnetite materials. For the quantitative analysis of the coexistence of different iron oxide phases in NPs, X-ray absorption spectroscopy (XAS) and X-ray magnetic circular dichroism spectroscopy (XMCD) have been used to quantitatively estimate the compositions of the mixture of maghemite and magnetite (γ-Fe_2_O_3_)_1−x_(Fe_3_O_4_)_x_, with the value of x ranging from 0.20 to 1.00 [56]. In addition to spectroscopic techniques, magnetic properties are also measured to characterize magnetite NPs. For example, magnetization versus temperature measurements reveal a kink at low temperature related to the Verwey transition of Fe_3_O_4_, while this feature does not occur in γ-Fe_2_O_3_. The Verwey transition in Fe_3_O_4_, which is usually observed for bulk magnetite at approximately 115–124 K, is also a good parameter to evaluate the level of crystallinity and match the stoichiometry of Fe_3_O_4_ NPs [41,96]. Hence, a combination of different characterization techniques is important to identify important factors that affect the magnetic properties of Fe_3_O_4_ NPs, such as the existence of the material phases, the uniformity of compositions, and the crystalline level.

## 4. Applications of Fe_3_O_4_ Nanoparticles

### 4.1. Biomedical Applications (Therapeutic and Diagnostic Technologies)

Combinations of excellent magnetic properties, inexpensive materials, great biocompatibility, and iron oxide have been widely investigated in various medical applications [35]. FDA approval for employing iron oxide nanoparticles as medical contrast agents for magnetic resonance imaging (MRI) indicates significant progress in applying magnetic iron oxide nanoparticles. Conventionally, magnetic contrast agents, hyperthermia, and drug delivery are the three most frequently explored biomedical applications of Fe_3_O_4_ nanoparticles. Furthermore, surface functionalization or preparation of bioconjugated Fe_3_O_4_-based substances has provided additional applications. We will introduce applications of Fe_3_O_4_ nanoparticles and their functionalized or core–shell structures in MRI, hyperthermia, and drug delivery. Moreover, we will highlight recent efforts in the preparation of multifunctional nanoparticles that integrate multiple applications in a single Fe_3_O_4_ nanoparticle-based system.

#### 4.1.1. Contrast Agents for Magnetic Resonance Imaging (MRI)

Magnetic contrast agents for MRI are some of the most promising applications of iron oxide nanoparticles. Several commercial iron oxide nanoparticles are used as MRI contrast agents, such as Feridex (dextran-coated Fe_3_O_4_ and γ-Fe_2_O_3_), Resovist (carboxydextran-coated Fe_3_O_4_), and Combidex (dextran-coated Fe_3_O_4_) [97–99]. Contrast agents have been used to enhance the contrast and improve the resolution of diagnostic MRI by reducing either the longitudinal (T_1_) or transverse (T_2_) relaxation time of water protons. Consequently, two types of contrast agents, T_1_ and T_2_, enhance the positive (bright signal) or negative (dark signal) contrast, respectively. As presented in Equations (1) and (2), the inverse of the relaxation time (1/T_1_ and 1/T_2_) is called the relaxation rate and is plotted as a function of the iron concentration to obtain linear lines. From those data, we are able to determine the slopes that are relaxivities r_1_ and r_2_, respectively. The higher the value for either r_1_ or r_2_, the better the contrast performance of T_1_ or T_2_ contrast agents, respectively. In addition, the ratio of relaxivities, r_2_/r_1_, can be used to evaluate the potential of a contrast agent, with lower values preferred for T_1_ agents and higher values for T_2_ agents. Although Fe_3_O_4_ nanoparticles have been extensively investigated as T_2_ contrast agents [100–104], increasing efforts to use Fe_3_O_4_ nanoparticles as T_1_ contrast agents have been reported recently [98,105]. In-depth mechanisms of T_1_-weighted and T_2_-weighted contrast agents in MRI and criteria for NPs as efficient MRI contrast agents were discussed in previous reviews [97,98]. These investigations were conducted with both superparamagnetic and ferrimagnetic Fe_3_O_4_ NPs, usually including functionalized NPs or core–shell architectures for better colloidal stability and biocompatibility.


(1) $$\frac{1}{{\text{T}}_{1}}=\frac{1}{{\text{T}}_{1}([\text{Fe}]=0)}+{\text{r}}_{1}[\text{Fe}]$$

(2) $$\frac{1}{{\text{T}}_{2}}=\frac{1}{{\text{T}}_{2}([\text{Fe}]=0)}+{\text{r}}_{2}[\text{Fe}]$$

**Figure 8.**
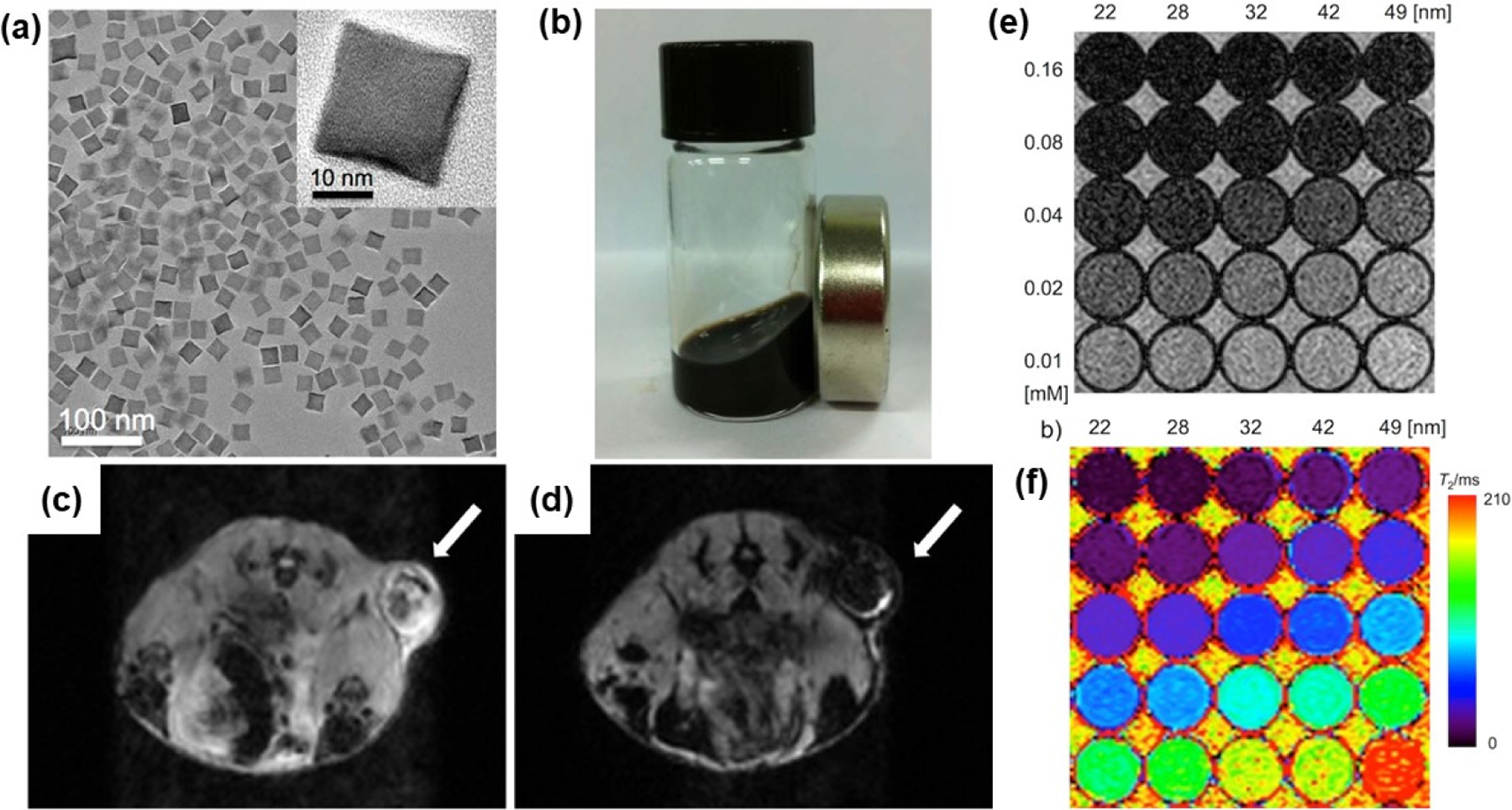
(**a**) TEM image of 22 nm nanocubes encapsulating PEG-phospholipid. (**b**) Image of colloidal iron oxide nanocubes. In vivo MR images of the tumor site: (**c**) without colloid injection and (**d**) after 1 h (intravenous injection). MR contrast effect of ferrimagnetic iron oxide nanocubes with different sizes: (**e**) T_2_-weighted MR images obtained with various concentrations of iron in a 3 T field and (**f**) their color-coded presentation. Reproduced with permission from ref. [106]. Copyright 2012 American Chemical Society.

Different geometries, sizes, and nanostructures of Fe_3_O_4_-based nanoparticles have been investigated as MRI contrast agents. Lee et al. prepared ferrimagnetic uniform-sized Fe_3_O_4_ nanocubes with an edge length of 22 nm functionalized with PEG-phospholipids to provide excellent colloidal stability in aqueous media and great biocompatibility [106]. The colloid-containing functionalized 22 nm-sized Fe_3_O_4_ nanocubes exhibit a very high r_2_ relaxivity of 761 mM^−1^ s^−1^, which achieved the theoretically predicted maximum r_2_ relaxivity. Figure 8a–d present the morphology of nanocubes, an image of a stable colloid, and in vivo images of tumors visualized with and without nanocube contrast agents. T_2_-weighted images produced with different sizes of nanocubes in various concentrations and their color-coded images are shown in Figure 8e,f. Separately, hyperbranched polyglycerol-grafted Fe_3_O_4_ NPs (size from 6 to 9 nm) were successfully synthesized with excellent colloidal stability in water, phosphate-buffered saline (PBS), and cell culture medium [107]. These NPs exhibit a contrast enhancement effect on T_2_-weighted sequences. Moreover, highly crystalline 8 nm Fe_3_O_4_ NPs were functionalized with an epoxy silane, (3-glycidyloxypropyl) trimethoxysilane, followed by the use of three-membered ring-opening chemistry to further graft polymers (e.g., polyetheramine and arginine) to the particle surface [108]. These functionalized NPs were highly dispersible in water and exhibited a high spin–lattice relaxivity r_1_ of 17 s^−1^ mM^−1^ and low r_2_/r_1_ ratios ranging from 3.3 to 3.8, which are good characteristics for T_1_ positive contrast agents. Bai and coworkers synthesized ultrasmall superparamagnetic Fe_3_O_4_ NPs modified with bull serum albumin and investigated these NPs as T_1_–T_2_ dual-modal MRI contrast agents in rabbit hepatic tumors [109]. In vivo experiments showed a rapid T_2_-weighted effect after 5 min, and T_1_ contrast enhancement appeared 90 min after intravenous administration. An Fe_3_O_4_-based dual-mode contrast agent for MRI and computed topography (CT) imaging was developed by Dheyab et al. [110]. In this study, Fe_3_O_4_@Au core–shell nanoparticles with a size of approximately 21 nm were prepared using a simple sonochemical method. The obtained core–shell NPs showed transverse relaxivity values of 222.28 mM^−1^ s^−1^ and have good potential for applications in MRI and CT imaging.

#### 4.1.2. Magnetic Hyperthermia

Magnetic hyperthermia has been documented as a promising therapeutic approach in cancer treatment. During these treatments, magnetic NPs provide heat to kill tumor cells in the presence of an alternating current (AC) magnetic field [34,111]. Tumor cells are destroyed upon the application of heat at 43 °C to 46 °C for a particular duration, while healthy cells are less affected by these treatments [112,113]. For safe clinical requirements, the product of the AC field amplitude and frequency should be smaller than 5 × 10^9^ Am^−1^ s^−1^ [114]. Hence, Fe_3_O_4_ nanoparticles must meet two criteria to be utilized as an efficient hyperthermal agent. First, they should achieve high heating efficiency under the safe range of the AC field with a small dose of MNPs. Second, nanoparticles should form stable colloids that allow intravenous injection or delivery to tumors via the blood-stream. In addition, Fe_3_O_4_ NPs should be functionalized to enhance biocompatibility and colloidal stability or conjugated with recognition molecules for specific target molecules. Superparamagnetic NPs potentially represent a good candidate because they exhibit good dispersion and a lack of aggregation due to their negligible coercivity values and remnant magnetization. In contrast, SPM NPs can suffer from low heating power and require a high dose of materials due to low saturation magnetization. Although ferrimagnetic NPs exhibit high heating efficiency, stable colloids of these NPs are difficult to prepare due to the strong magnetic dipole interactions of ferromagnetic particles. The mechanism of heat generation is governed by the hysteresis loss mechanism for ferro- and ferrimagnetic NPs [35]. Meanwhile, Néel and Brown relaxations are the main heating mechanisms for superparamagnetic NPs. The specific absorption rate (SAR), also called the specific loss power (SLP), is measured by calculating the rate of absorbed energy per unit of mass in W/g to evaluate the heating efficiency of NPs.

Numerous studies have investigated the effects of the size, geometry, and anisotropic properties of magnetite NPs on their hyperthermia performance under different AC magnetic field conditions. In one study, 26 nm Fe_3_O_4_ spheres (diameter) and cubes (edge) prepared using solvothermal methods exhibited a much higher SAR value, especially when increasing the magnetic field amplitude [71]. A more comprehensive study of the effects of the Fe_3_O_4_ nanocrystal sizes and geometries on heating efficiency was conducted by Nemati et al. [41]. Spherical and cubic Fe_3_O_4_ NPs with sizes ranging from 10 to 100 nm were synthesized, and their heating efficiency was evaluated. At 800 Oe and 310 kHz, the optimum sizes of nanospheres for hyperthermia range from 30 to 50 nm (650 W/g), and nanocubes with sizes ranging from 30 to 35 nm are better heaters. Furthermore, the study suggested that nanocubes supply more heat than nanospheres with a size smaller than 35 nm, while nanospheres with a size larger than 35 nm are better heating agents. The hyperthermia performance of Fe_3_O_4_ nanocubes with sizes ranging from 13 to 40 nm was recorded at different magnetic field amplitudes from 2.3 to 30 kAm^−1^, with three different frequencies of 320, 520, and 720 kHz [52]. The 19 nm cubes show the highest SAR values at all frequencies, which can reach 2452 W/g at 520 kHz and 29 kAm^−1^. Additionally, studies of the magnetic properties showed that 19 nm nanocubes are located at the transition point from superparamagnetic to ferrimagnetic, consistent with the hypothesis proposed in previous studies for the higher SAR value of iron oxide nanoparticles in this transition size range [115]. Muro-Cruces and coworkers also compared the heating efficiency of nanocubes with sizes of 13, 15, and 19 nm and 22 nm spherical NPs at 17 kA/m and 183 kHz and obtained the best heating efficiency for 19 nm cubic Fe_3_O_4_ nanocrystals [74]. However, other measurements were conducted at different frequencies, such as 109, 220, and 300 kHz, for a similar size range (14 to 35 nm) of Fe_3_O_4_ nanocubes and produced a slightly different result [44]. Nanocubes with sizes of 19 and 24 nm showed the best SAR value under most of the measured conditions and saturated at approximately 18 kAm^−1^; however, 35 nm cubes showed higher SAR values at magnetic field amplitudes of 20–24 kAm^−1^. Das et al. synthesized Fe_3_O_4_ nanorods, nanocubes, and nanospheres with comparable volumes and determined that nanorods achieve a higher SAR value of 862 W/g, which is much higher than nanocubes (314 W/g) and nanospheres (140 W/g) [40]. Furthermore, increasing the aspect ratio of nanorods was proven to help increase the SAR value of magnetite nanorods. Thus, the heating efficiency of magnetite nanoparticles is affected by the shape and size of the nanoparticles and remarkably depends on the frequency and amplitude of the applied AC magnetic field.

**Figure 9.**
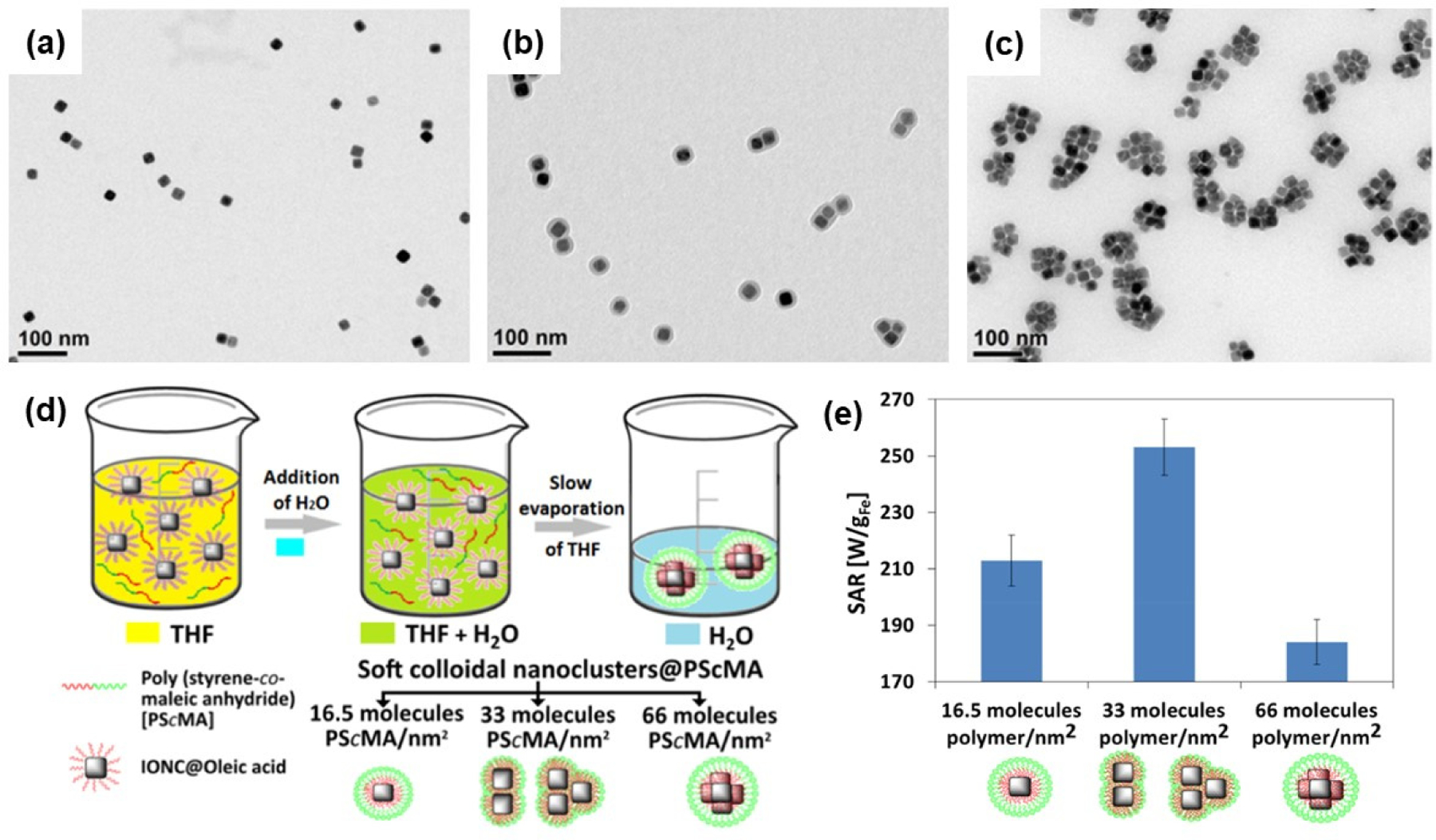
TEM images of colloidal nanoclusters constructed from Fe_3_O_4_ nanocube-coated amphiphilic copolymer poly(styrene-comaleic anhydride) to form (**a**) monomer clusters (1 nanocube), (**b**) dimers and trimers (2–3 nanocubes), and (**c**) centrosymmetric clusters (more than 4 nanocubes). (**d**) Schematic illustration of the preparation of soft colloidal nanoclusters. (**e**) SAR values for different soft colloidal nanoclusters. Reproduced with permission from ref. [117]. Copyright 2017 American Chemical Society.

Functionalization of Fe_3_O_4_ NPs or core–shell architectures was studied to further enhance the heating performance, biocompatibility, and stability of colloids. Bae et al. synthesized chitosan oligosaccharide-coated packs of four to ten 30 nm-sized Fe_3_O_4_ nanocubes and investigated the hyperthermia activity [116]. These particle cluster-coated chitosan nanoparticles exhibited a superior magnetic heating ability with a high specific loss power of 2614 W/g, which is much higher than that of commercial Feridex nanoparticles (83 W/g). Chitosan oligosaccharide was used to improve colloidal stability and blood circulation in vivo. Continuing the particle clustering strategy to enhance magnetic hyperthermia, Niculaes et al. studied the effect of individual nanocubes, dimers or trimers (two or three nanocubes), and centrosymmetric clusters (more than four nanocubes) on SAR values [117]. TEM images of monomers, dimers, trimers, and centrosymmetric clusters are shown in Figure 9a–c. The prepared nanocubes stabilized with oleic acid were coated with the amphiphilic copolymer poly(styrene-comaleic anhydride). The degree of clustering was controlled by the ratio of polymer/surface area of nanocubes, as illustrated in Figure 9d. The SAR data shown in Figure 9e indicate that the dimers and trimers exhibit the best heating efficiency. In another study, Zyuzin et al. confined nanocubes inside submicrometer cavities to preserve magnetic heat losses in an intracellular environment [118]. Another study of 2D assemblies of magnetic nanocubes exploited enzymatic polymer disassembly to improve magnetic hyperthermia heat losses [119].

Interestingly, Espinosa et al. utilized 20 nm Fe_3_O_4_ nanocubes with a dual capacity to act as both magnetic and photothermal agents [120]. Upon exposure to an AC magnetic field and near-infrared laser irradiation, an aqueous suspension of magnetite nanocubes achieved an unprecedented heating power up to 5000 W/g. The dual mode of magnetic hyperthermia and photothermia resulted in complete apoptosis-mediated cell death and complete solid tumor regression in vivo. Lavorato et al. synthesized monodisperse core–shell nanoparticles Fe_3_O_4_@Co_x_Zn_1−x_Fe_2_O_4_, and by controlling the thickness and composition of the shell, the water colloid of these NPs exhibited a large heating power up to 2400 W/g under an 80 mT magnetic field with a frequency of 309 kHz [121]. Lak and coworkers studied the phase transformation of 23 nm FeO@Fe_3_O_4_ core–shell nanocubes and their magnetic heating performance. This study identified the roles of subdomains of FeO, Fe^2+^ deficiencies, and structural defects in the up to 10-fold increase in the magnetic losses of the nanocubes, resulting in excellent heating efficiency compared to pure magnetite phase nanocubes [122]. Therefore, studies have shown that the sizes, geometries, and nanostructures of Fe_3_O_4_ NPs are three main factors that determine their magnetic heating capability.

#### 4.1.3. Drug Delivery

An increasingly applied solution for the efficient utilization of drugs against target pathogens with minimum doses is drug delivery. Fe_3_O_4_ NPs are used as drug carriers in drug delivery applications due to the combinations of their multiple valuable properties, such as a strong magnetic response, low toxicity, biodegradability, biocompatibility, and inexpensiveness [123]. Furthermore, facile syntheses, easily functionalized surfaces for bioconjugate coatings or enhanced colloidal stability, and the capability to be guided under a magnetic field make Fe_3_O_4_ NPs an excellent candidate for drug delivery. In addition, the pristine magnetic properties for MRI imaging or hyperthermia of Fe_3_O_4_ can be integrated to achieve multifunctional NPs for drug delivery. Fe_3_O_4_ nanocarriers are usually functionalized with polymers (e.g., pH-responsive polymers) to control drug release, biomolecules for targeting ligand–receptor species, and biocompatible porous shells (e.g., SiO_2_) for drug loading. Cheng et al. prepared 16 nm porous hollow Fe_3_O_4_ NPs with opening pores of 2–4 nm for cisplatin (an anticancer drug) storage and release [87]. The release rate of the anticancer drug cisplatin increased further at pH values lower than 6 due to acidic etching of the NP pores. These NPs were also coupled with Herceptin to target breast cancer cells. Poly(acrylic acid) (PAA), a pH-responsive polymer, was loaded into hollow 200 nm-diameter Fe_3_O_4_ NPs to obtain the pH-triggered and magnetic-targeted drug delivery of the anticancer drug doxorubicin [124]. Moreover, rattle-type Fe_3_O_4_@SiO_2_ hollow mesoporous spheres were synthesized with different sizes and different thicknesses of the mesoporous shell using carbon templates [125]. These NPs showed no toxicity up to a concentration of 150 μg/mL and were able to load the anticancer drug doxorubicin hydrochloride into their hollow mesopores. Folic acid, a receptor-specific ligand for the targeted delivery of anticancer drugs, was further loaded on rattle-type Fe_3_O_3_@SiO_2_ hollow mesoporous spheres to achieve a combination of receptor-mediated targeting and magnetic targeting [126]. Qiu and coworkers introduced the ZnO interlayer as a microwave absorber, forming a novel Fe_3_O_4_@ZnO@SiO_2_ drug carrier that controls the release of cancer drugs upon exposure to microwave irradiation [127]. Special Janus nanocomposites with dual surface functionalization of polystyrene@Fe_3_O_4_@SiO_2_ were synthesized by Wang et al. for tumor cell targeting and stimulus-induced drug release [128]. Based on these examples, Fe_3_O_4_ nanoparticles with magnetic properties, facile synthesis of hollow nanostructures, and easily functionalized surfaces are promising platforms for drug delivery applications.

#### 4.1.4. Multifunctional Nanoparticles in Biomedical Applications

The potential of applying Fe_3_O_4_ NPs in biomedical applications is not limited to single functional agents, such as contrast agents, heating agents (hyperthermia), or drug carrier nanospecies. Surface functionalization and core–shell structures of Fe_3_O_4_ NPs achieve multifunctional nanoagents with maximal activity in biomedical applications that integrate both therapeutic and diagnostic technologies in an Fe_3_O_4_ NP-based system. For example, MRI contrast agents and hyperthermia agents based on Fe_3_O_4_ nanostructures have also been used for drug delivery or integrated with biological substances and fluorescent molecules to enhance functionality in disease treatment and imaging. Immobilization of human tissue plasminogen activator (tPA) and bovine serum albumin on a cluster of multiple 20 nm Fe_3_O_4_ nanocubes was reported by Voros et al. for the preparation of multifunctional thrombolytic and MRI contrast nanoagents [129]. These nanoagents exhibited excellent thrombolytic activity and can potentially be applied for imaging vascular thrombi. The dissolution rate of clots was increased approximately 100-fold compared to free tPA due to the intimate interaction of tPA with the fibrin network and achieved an additional 10-fold increase through localized heating upon exposure to an alternating magnetic field. Lai and coworkers synthesized Fe_3_O_4_@SiO_2_ core–shell nanoparticles integrated with phosphorescent iridium complexes for three-in-one purposes of MRI, luminescence imaging, and photodynamic therapy [130]. Highly uniform superparamagnetic Fe_3_O_4_ NPs with an average size of 12 nm were coated with porous, biocompatible SiO_2_ shells before the iridium complexes were incorporated into the SiO_2_ matrix. Phosphorescent iridium complexes serve as dual functional agents, including photosensitizers, to generate singlet oxygen (^1^O_2_) for inducing cancer cell apoptosis and as luminescence agents for luminescence imaging. In a recent study aiming to prepare a dual-modality magnetic resonance and fluorescence unified imaging platform performed by Bao and colleagues [131], lipid-encapsulated Fe_3_O_4_ NPs were combined with dialkylcarbocyanine dyes before being integrated with surface peptide bioconjugation. The nanoprobes were used for stable, high-contrast MRI scans, near-infrared fluorescence imaging, and fluorescence microscopy.

**Figure 10.**
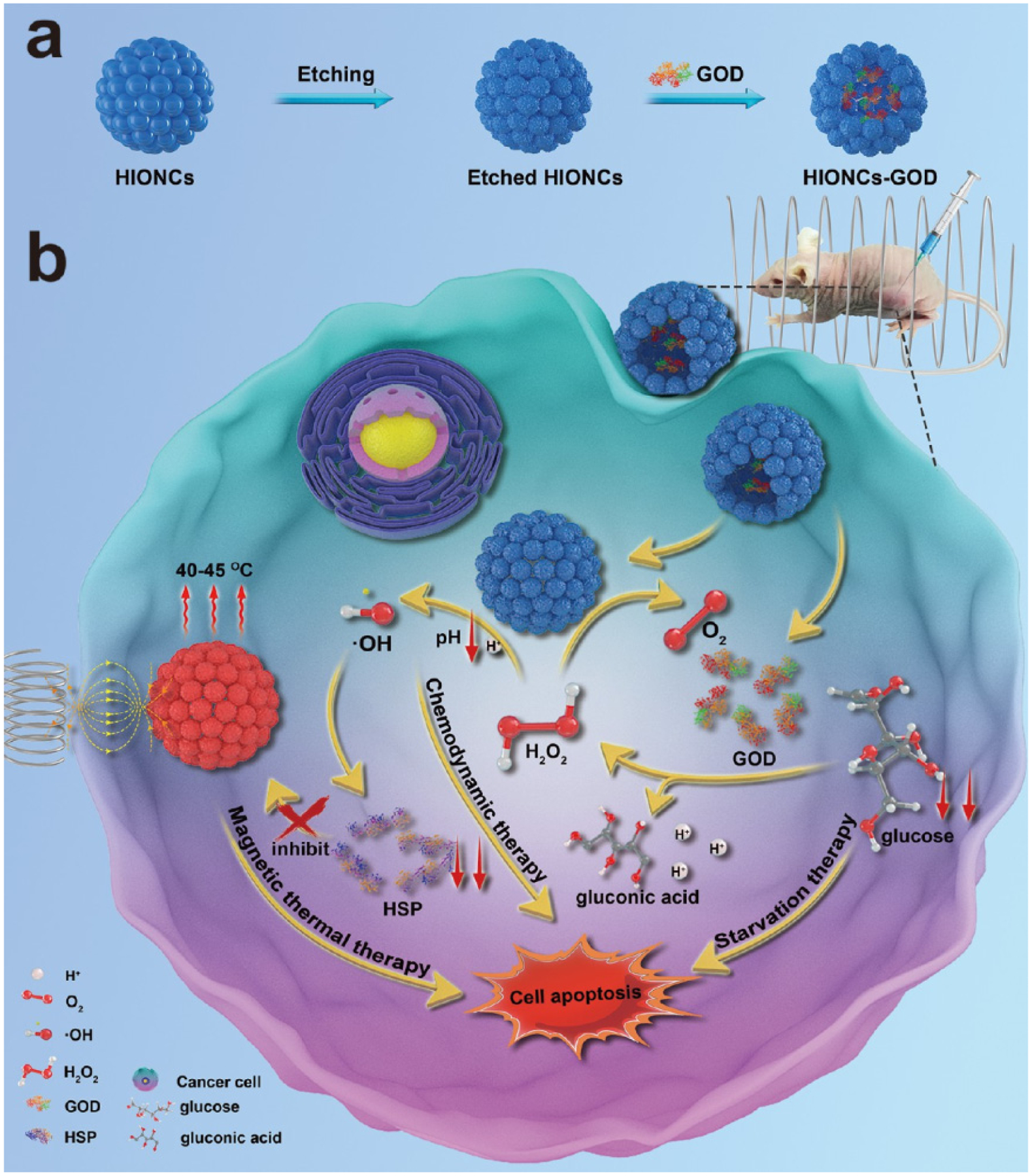
(**a**) Synthetic scheme for GOD-encapsulated hollow iron oxide nanoparticles and (**b**) multifunctional therapeutic strategies for starvation–chemodynamic–hyperthermia using GOD-encapsulated hollow IONPs. Reproduced with permission from ref. [89]. Copyright 2020 American Chemical Society.

A novel magnetic nanocatalyst constructed from glucose oxidase (GOD)-loaded Fe_3_O_4_ hollow NPs as starvation–chemodynamic–hyperthermia synergistic therapy for tumors was reported by Ying et al. [89]. Figure 10 illustrates the working principle of the nanocatalysts. Ferrous cations generate the reactive oxygen species (ROS) OH radicals from H_2_O_2_ via the Fenton reaction and subsequently induce cell apoptosis by chemodynamic therapy. GOD consumes glucose, which is an important nutrient in tumor tissues, resulting in tumor tissue starvation during therapy and the generation of an excess amount of H_2_O_2_ to further enhance the Fenton reaction in terms of chemodynamic activity. Moreover, GOD-loaded hollow Fe_3_O_4_ NPs can be heated under an AMF for hyperthermia treatment. In addition to utilizing Fe_3_O_4_ nanoparticles as the main core for further functionalization purposes, active MRI and hyperthermic Fe_3_O_4_ NPs have been grafted onto the surface of Er^3+^/Yb^3+^-doped NaYF4@SiO2@AuNP core–shell nanoparticles to obtain near-infrared and magnetic-responsive nanocomposites for hyperthermia treatment [132]. Combining an active optical heater Er^3+^/Yb^3+^-doped YPO_4_ nanophosphor with magnetic hyperthermia Fe_3_O_4_ agents into a hybrid material Er^3+^/Yb^3+^-doped YPO_4_@Fe_3_O_4_ enhances the hyperthermic activity and ability to recover the material [133].

### 4.2. Biosensing

Magnetic nanoparticle-based solutions for biosensing have been widely studied, and significant progress has been achieved [7]. In general, magnetic NPs have been used for labeling magnetic signals for various biological sensing purposes such as biomolecular detections based on ligand–receptor binding of biological substances. In these systems, the magnetic NPs are functionalized for facile conjugation with biomarkers. The magnetic signals can be detected by different magnetic detection techniques including spintronic sensors, nuclear magnetic resonance sensors, atomic magnetometer-based sensors, and superconducting quantum interference devices. Various types of magnetic responses can be used for detection depending on the sensing purposes and detection techniques such as relaxation, remnant magnetization, susceptibility, induced ^1^H NMR, and frequency mixing. In-depth methods for functionalization of magnetic particles, strategies for bioconjugation, and designs of magnetic detection for magnetic particle-based biosensing platforms were summarized in the reviews of Chen et al. [7] and Hsing et al. [134].

**Figure 11.**
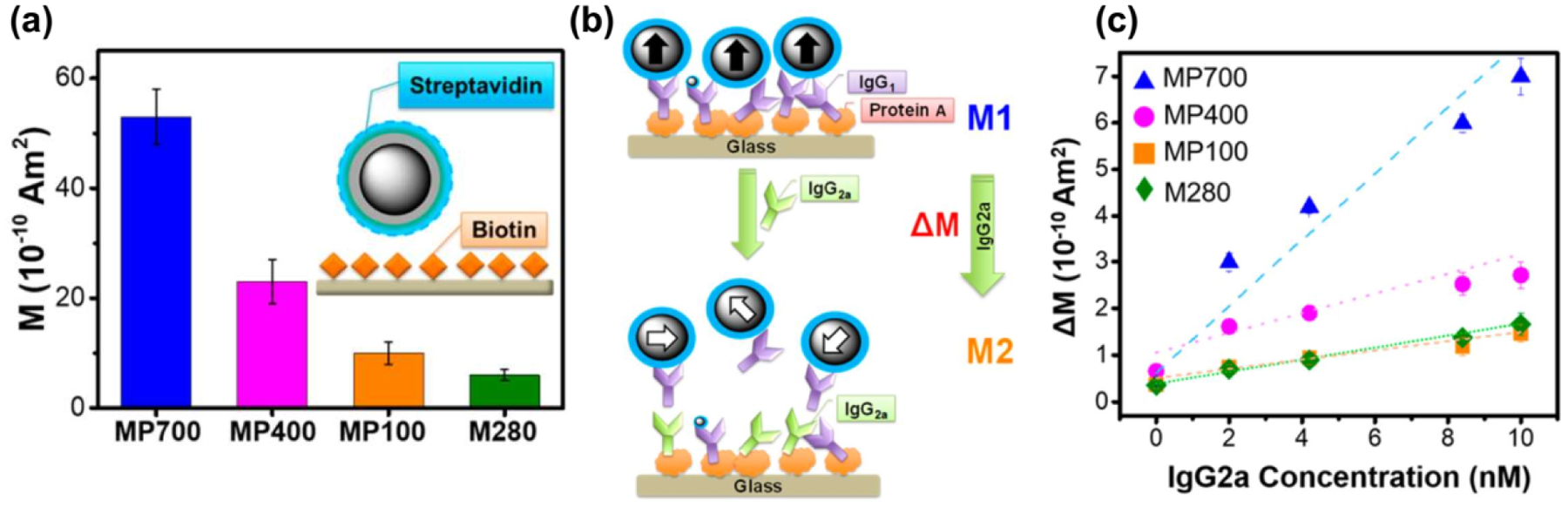
(**a**) Magnetic signal of streptavidin-conjugated Fe_3_O_4_@SiO_2_@PAA NPs bound to a biotinylated surface. (**b**) Schematic illustration of the interaction between magnetic NPs and the surface for protein detection in the EXIRM analysis. (**c**) EXIRM data for Protein A arising from an exchange between IgG1 and IgG2 subclasses. Reproduced with permission from ref. [43]. Copyright 2018 American Chemical Society.

Due to their strong magnetic properties, biocompatibility, and facile functionalized surface, Fe_3_O_4_ nanoparticles have been utilized in various biosensor platforms [135–138]. Various biosensing platforms utilizing Fe_3_O_4_ NPs were summarized in the review by Kim et al. [139]. The magnetic properties, sizes, morphologies, and crystallinity of NPs are important factors defining the performance of Fe_3_O_4_ nanoparticle-based sensing systems. Lee and colleagues examined the biosensing performance of Fe_3_O_4_ nanoparticles from two aspects: the sizes and geometries of NPs (cubes and spheres) [42,43]. As an approach to study the size effect of NPs in biosensing, three different sizes of 120, 440, and 700 nm Fe_3_O_4_ nanospheres with comparable degrees of crystallinity (grain size ~17–18 nm) were synthesized, coated with SiO_2_, functionalized with poly(acrylic) acid (PAA) before conjugation with streptavidin, and utilized to detect specific proteins. As shown in Figure 11a, larger spherical NPs exhibit better magnetic signals and surprisingly better performance than commercial magnetic beads (2.8 μm size) in an experiment detecting the well-known streptavidin–biotin interaction. In addition, the streptavidin-conjugated magnetic NPs were further combined with an exchange-induced remnant magnetization (EXIRM) platform for the specific detection of two immunoglobulins G (IgG1 and IgG2a) bound to Protein A. Figure 11b shows a schematic illustrating protein-immunoglobulin exchange detection using EXIRM measurements. Protein A has a stronger affinity for IgG2a than IgG1. Therefore, Protein A is immobilized covalently on the glass surface and then attaches to biotinylated IgG1, followed by the attachment of streptavidin-coated NPs to bound biotinylated IgG1. With stronger affinity for Protein A, IgG2a is then introduced, causes an exchange reaction, and influences the magnetization signal. Figure 11c shows that larger MPs exhibit larger slopes, and magnetic NPs with a size of 120 nm show equivalent signals to commercial magnetic beads. The combination of the larger size and strong magnetic properties of functionalized NPs increases the sensitivity by up to 9-fold compared with commercial magnetic beads and achieves a high detection specificity.

**Figure 12.**
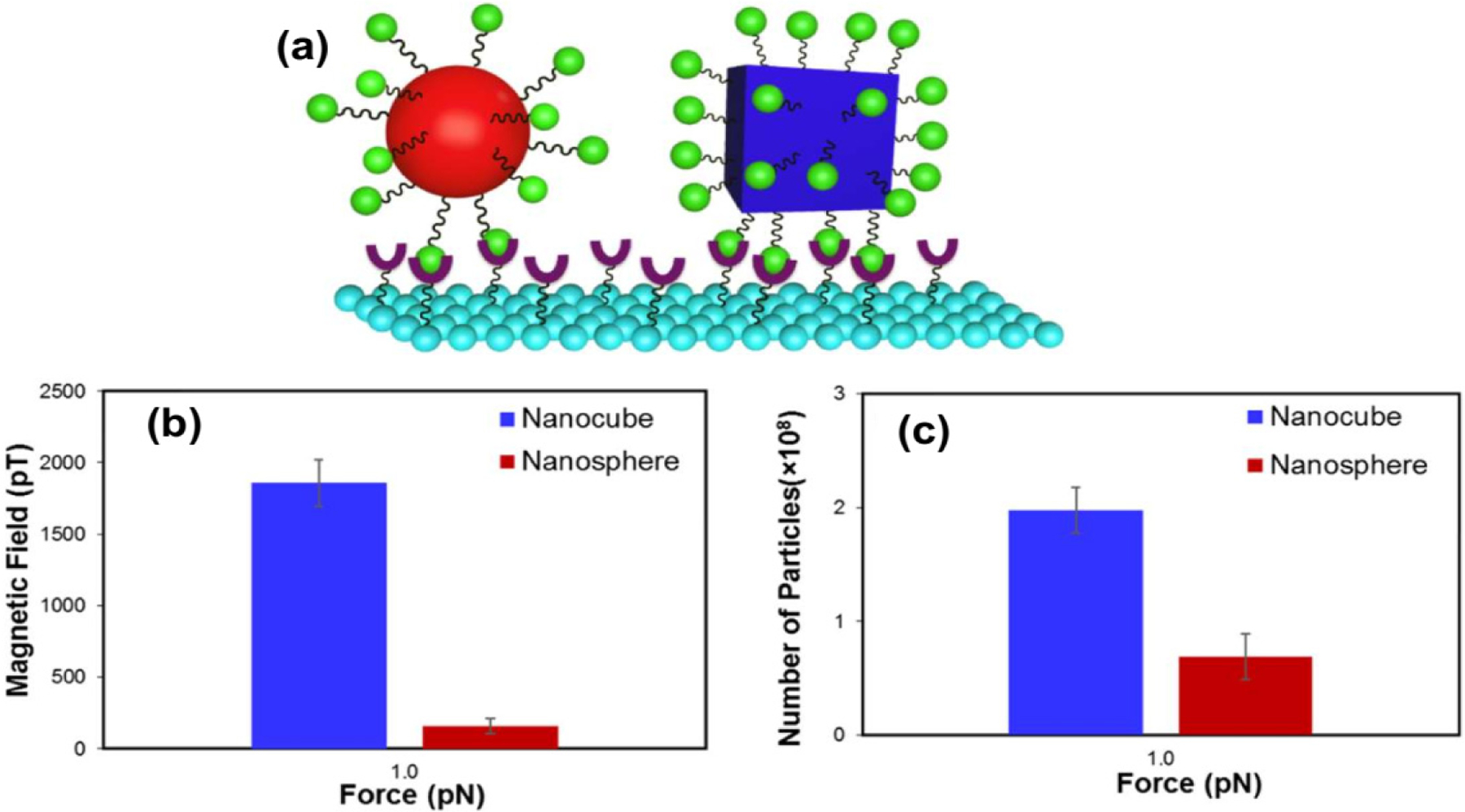
(**a**) Schematic illustration of the contact area in the biosensing of nanocubic and nanospherical Fe_3_O_4_. FIRMS data showing the (**b**) magnetization profiles versus applied force of nanocubes and nanospheres with similar volumes and (**c**) number of retained nanoparticles on the sensor at 1 pN. Reproduced with permission from ref. [42]. Copyright 2017 American Chemical Society.

In addition to the importance of appropriate sizes in biosensing, the morphology of NPs has been identified as an important parameter for efficient biosensing performance. Kolhatkar et al. synthesized spherical and cubic Fe_3_O_4_ NPs with multiple domains and sizes ranging from 100 to 225 nm and studied their crystallinity, magnetic properties, and biosensing performance using force-induced remnant magnetization force spectroscopy (FIRMS). By comparing the magnetic properties of same-volume and same-body diagonal/diameter nanoparticles, cubic NPs exhibited 1.4–3.0 and 1.1–8.4 times higher values for saturation magnetization (M_S_) and coercivity (H_C_), respectively. The structural analysis revealed that the high crystallinity of nanocubes explained the enhanced magnetic properties. The author also functionalized these NPs with biotin and used FIRMS to study their binding to the streptavidin-modified surface. Upon applying a 1 pN force, the number of particles remaining on the functionalized surface and the magnetization response of the nanocubes were much better than those of the nanospheres, as indicated in Figure 12b,c. Thus, the increase in the signal and stronger attachment to the surface can be explained by the difference in magnetic strength and the contact surface area (Figure 12a).

### 4.3. Environmental Applications

Fe_3_O_4_ nanoparticles and their nanoarchitectures have been utilized for applications in environmental treatments, such as heavy metal removal and adsorption/degradation of organic pollutants [18]. In these applications, Fe_3_O_4_ NPs have been used as supporting platforms that can easily be recovered/recycled by using magnetic separation techniques. Inexpensiveness, easy fabrication, biocompatibility, and easy separation/recovery by magnetic fields are the advantages of using Fe_3_O_4_ nanoparticles in environmental applications. However, easy agglomeration (for ferrimagnetic NPs) and oxidation are drawbacks that can be overcome by applying an appropriate surface coating layer or functionalized surface. The surface coating layer should be stable under harsh chemical conditions (e.g., acidic and basic conditions), have high thermal and mechanical stability, and be porous with a high loading capacity for pollutants. In general, magnetically loaded adsorbents are usually core–shell structures or composites of Fe_3_O_4_ with other oxides (e.g., SiO_2_, TiO_2_), carbon-based materials (carbon, carbon nanotubes, and graphene), and polymers or organic molecules with a good affinity for heavy metals or organic pollutants. Zhang et al. synthesized superparamagnetic Fe_3_O_4_@C core–shell nanoparticles with an average size of ~250 nm and studied the adsorption kinetics of two dyes, methylene blue (MB) and cresol red (CR) [140]. The prepared magnetic NPs are dispersible in an aqueous solution, easily separated from the solution using an external magnet, and have adsorption capacities for MB and CR of 44.38 mg/g and 11.22 mg/g, respectively. Separately, carboxylatopillar [5] arene-modified Fe_3_O_4_ nanoparticles synthesized using a one-pot solvothermal technique exhibit excellent cationic dye separation over a wide range of pH values and can be recycled by simple washing without decreasing the adsorption properties [141]. Khalaf examined the ability of Fe_3_O_4_ nanocubes coated with SiO_2_ and TiO_2_ to remove Cr(VI) [142]. Ren and coworkers also prepared ferrimagnetic Fe_3_O_4_@carbon composites and studied their performance in both removing the heavy metal Cr(VI) and organic pollutant Congo red [143]. Furthermore, Fe_3_O_4_-functionalized nanoparticles and composites have been applied to remove various heavy metal cations, such as Pb(II), Cd(II), Cu(II), Hg(II), As(III), and Cr(III) [144–150], and organic pollutants, including rhodamine B, rhodamine 6G, methyl orange, and oil [151–153]. Additionally, Fe_3_O_4_ NPs have been used as magnetic carriers for photocatalyst materials (e.g., TiO_2_) to promote the photocatalytic degradation of organic pollutants such as methylene blue and ofloxacin fluoroquinolone [154]. In general, Fe_3_O_4_ nanoparticles function as efficient magnetic carriers for absorbents or photocatalysts, which provide recyclability, reusability, nontoxicity, and inexpensive materials for large-scale wastewater treatments.

### 4.4. Energy Conversion and Storage Devices

Due to their various advantageous characteristics, such as low cost, natural abundance, ecofriendliness, electrochemical activity, and high theoretical capacity, Fe_3_O_4_ is a potentially useful anode material for supercapacitors and lithium-ion batteries. With good conductivity (10^2^–10^3^ Ω^−1^ cm^−1^) and a high theoretical capacitance (approximately 347 F/g at 1.2 V), Fe_3_O_4_ has been used in high-energy-density storage supercapacitors via redox reactions [20,155]. Nanostructured materials constructed from Fe_3_O_4_ NPs and carbon-based materials are promising hybrid materials that achieve a high energy density and robust electrochemical performance. Several hybrid nanostructures, including a 3D network of Fe_3_O_4_ NPs/reduced graphene oxide nanosheets [156], Fe_3_O_4_@carbon nanosheets [157], Fe_3_O_4_ nanosphere-decorated graphene [158], and Fe_3_O_4_ nanospheres coated with nitrogen-doped carbon [159], have produced encouraging results in the construction of high-performance supercapacitors. Using Fe_3_O_4_ as a conductive core, nanocomposites of conductive ferroelectric core–shell Fe_3_O_4_@BaTiO_3_ nanoparticles loaded into the ferroelectric poly(vinylidene fluoride-cohexafluoropropylene) (P-(VDF-HFP)) polymer matrix were prepared to increase the permittivity of the polymer and increase the degradation resistance of the polymerbased capacitor. The maximum energy storage density of this thin film reaches 7.018 J/cm^3^ upon exposure to an electric field of 2350 kV/cm [160]. Similar core–shell Fe_3_O_4_@BaTiO_3_ NPs were also incorporated into a PVDF polymer matrix and exhibited a remarkable energy density storage of 16 J/cc under an electric field of 430 kV/mm [161]. In addition to capacitor/supercapacitor applications, Fe_3_O_4_ NPs have been integrated into various nanoarchitectures/nanocomposites for use as anode materials for rechargeable lithium-ion batteries. In these systems, Fe_3_O_4_ participates in an electrochemical reaction with lithium, as indicated in Equation (3) below [162], which allows Li cation insertion/extraction. Theoretically, the capacity of Fe_3_O_4_ can reach ~900 mA h g^−1^, which is even higher than the capacity of commercial graphite (~372 mA h g^−1^). Optimizing the sizes and shapes of nanoparticles [163–166] and fabricating carbon hybrid materials of Fe_3_O_4_ [167–170] efficiently increase the conductivity, structural integrity, and performance of devices. Graphene nanosheet-wrapped Fe_3_O_4_ particles prepared by Zhou and coworkers showed improved stability and an excellent rate [168]. Wei et al. designed 3D graphene foams (GFs) cross-linked with graphene sheet (GS)-encapsulated Fe_3_O_4_ nanosphere Fe_3_O_4_@GS/GF, which exhibited a high reversible capacity of 1059 mAh g^−1^ over 150 cycles [171]. Thus, due to its superior properties, Fe_3_O_4_ represents a potential oxide material for integration into energy conversion and storage devices.

Electrochemical reaction (3):

(3) $${\text{Fe}}_{3}{\text{O}}_{4}+8{\text{Li}}^{+}+8{\text{e}}^{-}↔3\text{Fe}+4{\text{Li}}_{2}\text{O}$$

## 5. Conclusions

This review provides updates on recent progress and covers important aspects of applied Fe_3_O_4_ NPs, including structures and synthesis, magnetic properties, strategies for functionalization, and performance of Fe_3_O_4_-based NPs in various emerging applications. The size/geometry–property relationship of NPs, size/geometry-application relationship, and role of nanostructures in target applications were highlighted. The structures of Fe_3_O_4_ NPs (sizes and geometries) and magnetic properties are two important features that could be tailored by applying appropriate synthetic strategies. Consequently, the sizes and geometries of magnetic Fe_3_O_4_ NPs are very important structural factors that determine many properties of nanoparticles and their capabilities in various applications. This review also provides a limited atlas for the selection of suitable synthetic methods to obtain appropriate sizes, geometries, and magnetic properties of Fe_3_O_4_ NPs for target applications. Moreover, recent progress in applying Fe_3_O_4_ nanoparticles in emerging applications, such as diagnostic and therapeutic applications in the biomedical, biosensing, environmental, and energy storage fields, has been summarized. The exploitation of an appropriate structure of Fe_3_O_4_ NPs with the desired properties combined with the additional contribution of coating materials, functionalization of the surface, and special nanoarchitectures are crucial strategies to increase the performance of Fe_3_O_4_ NPs and achieve multifunctional NPs. Thus, the exploration of Fe_3_O_4_ NPs with different sizes, geometries, and integrated functionalities for multiple applications is an interesting field of research that still retains endless opportunities for discovery.

## Figures and Tables

**Table 1.** Syntheses and magnetic properties of spherical Fe_3_O_4_ NPs with a size <25 nm in a superparamagnetic regime at room temperature.

Diameter (nm)	Precursor	Method	Size Control Factor	M_S_ (emu/g)^a^	Ref.
4 (seeds), 8, 12, 16	Fe(acac)_3_	Thermal decomposition	Seed-mediated growth	82 (for 16 nm)	[54]
6 (seeds), 8, 10, 16	Fe(acac)_3_	Thermal decomposition	Temperature, seed-mediated growth	83 (for 16 nm)	[55]
5, 9, 12, 16, 22	Fe(oleate)_3_	Thermal decomposition	Different organic solvents or concentration of surfactant	Unknown	[56]
7, 8, 9, 10	Fe(acac)_3_	Thermal decomposition	Ratio OAm/solvent. (OAm: surfactant and reducing agent)	76, 77, 79, 80	[57]
8, 11, 15, 18	Fe(acac)_3_	Thermal decomposition	Amount of surfactant	≈65–75	[58]
8, 11	Fe(acac)_3_	Solvothermal	Different surfactants	73.1, 109.4 (emu/g Fe)	[59]
4, 12 **60**	FeCl_3_·6H_2_O	Solvothermal	Reaction time	3, 59 **84 (ferri)**	[60]
15.4, 16.7, 22.4, 31.1	FeCl_3_·4H_2_O	Hydrothermal	Concentration of reactants and solvent composition	53.3, 65.1, 81.2, 97.4	[61]
4.2, 7.4, 8.1, 17, 45	Fe(acac)_3_	Thermal decomposition	Reducing agent, surfactant	75, 70, 65, 82, 92	[62]
6.6, 11.6, 17.8	FeCl_2_·4H_2_O FeCl_3_·6H_2_O	Solvothermal	Solvent composition	71, 77, 83	[63]
11	FeCl_2_·4H_2_O FeCl_3_·6H_2_O	Sonochemistry	None	80	[64]

a Saturation magnetization (M_S_) value at 300 K. **Bold** indicates that the size of NPs (diameter) exceeds 25 nm.

**Table 2.** Syntheses and magnetic properties of SNPs with sizes larger than 25 nm.

Diameter (nm)	Precursor and Reagents	Solvent	Size Control Factor	Grain Size (nm)	M_S_ (emu/g)	H_C_ (Oe)	[Ref.]
200, 400, 800	FeCl_3_·6H_2_O, NaAc, PEG	Ethylene glycol (EG)	Rxn time	UN	81.9 UN, UN	UN	[48]
31, 53, 71, 93, 141, 174	FeCl_3_, NaOH, PAA	Diethylene glycol (DEG)	NaOH stock solution	≈10	UN, 30.9, UN, 56.7, UN, 63.5	SPM	[53]
120, 190, 560 (SPs)	Fe_3_O_4_ NPs functionalized with OA and DTAB in chloroform. PVP in EG.		Concentration of DTAB and nanoparticles	5.8 (NPs)	UN	SPM	[67] [68]
280	FeCl_3_.6H_2_O Na(acrylate) NaAc	EG	-	5.9, 6.9, 8.3, 13.5	36.2, 38.7, 46.5, 67.2	SPM	[46]
6, 60, 120, 170		EG/DEG	Solvent composition	10	UN	SPM	
20, 90, 165, 300.	FeCl_3_·6H_2_O, NaAc, PVP	EG/DEG	Solvent composition	10–20	62.1, 62.1, 62.8, 63.9	8, *20, 28, 16*	[47]
82, 139, 188, 544, 728, 1116	FeCl_3_·6H_2_O, NaAc, PAA, H_2_O	EG	H_2_O	15.4, 20.7, 23.9, 18.6, 17.7, 17.6	*56, 71, 73, 79, 80,* 80.27	*115, 141, 149, 139, 136, 127*	[70]
100, 135, 150, 175, 275	FeCl_3_·6H_2_O, NaAc, PVP	EG	Rxn time	17, 17, 15, 12, 11	69, 72, 65, 32, 56	106, 42, 66, 21, 28	[42]
120, 440, 700	FeCl_3_·6H_2_O, NaAc, PEG	EG, DEG	Solvent composition, FeCl_3_	18, 17, 17	78, 84, 87	62, 73, 72	[43]

UN denotes unknown, as the information was not provided in the literature. SPM denotes superparamagnetic, H_C_ = M_R_ = 0 (at RT). Values in italics indicate that the data were estimated from figures provided in the referenced articles.

**Table 3.** Syntheses of Fe_3_O_4_ nanocubes and their magnetic properties.

Edge (nm)	Precursor and Reagents	Solvent(s)	Size Control Factor	M_S_ (emu/g)	H_C_ (Oe)	Ref.
9.3, 13.4, 15.5, 22.1	Fe(oleate)_3_ Sodium oleate	Octadecene, diphenyl ether, n-tetracosane	Surfactant, Temperature	UN	SPM	[73]
6.5 15 30	Fe(acac)_3_ 1,2-hexadecandiol Oleic acid, Oleylamine	Benzyl ether	Heat rate, Reaction time	39.5 80.5 83.0	SPM SPM 100 Oe	[49]
22 79 160	Fe(acac)_3_ Oleic acid	Benzyl ether	Concentration, Reaction time, Ligand	*152* *136* *144* emu/g(Fe)	*17* *88* *115*	[50]
13 45 67 100 124 180	Fe(acac)_3_ Decanoic acid	Benzyl ether	Ramping rate	54.7 89.9 89.0 92.8 86.0 81.9	SPM ≈*50* UN UN UN ≈*50*	[72]
12 19 25 38	Fe(acac)_3_ Decanoic acid	Benzyl ether	Degas temperature, Ramping rate	UN 80 UN UN	UN UN UN UN	[52]
14, 19, 24, 35	Fe(acac)_3_ Decanoic acid	Benzyl ether Squalene	Ramping rate, Solvent composition	64, 73, 75, 88	26, 28, 7, 23	[44]
22, 36, 57	Fe(acac)_3_ Mg(acetate)_2_ Sodium oleate Oleic acid	Benzyl ether	Sodium oleate	UN	UN	[58]
78, 87, 101, 130	Fe(acac)_3_ Oleic acid	Benzyl ether	Concentration, Reaction time	90, 95, 95, 80	139, 165, 177, 80	[42]
10–80	Fe(acac)_3_ Oleic acid Sodium oleate	Benzyl ether 1-octadene 1-tetradecene	Concentration, Degassing temperature, Ramping rate	84 (for 15.3 nm)	SPM (for 15.3 nm)	[74]
80	FeSO_4_·7H_2_O NaOH	H_2_O	None	85.8 emu/g	UN	[75]
26	Fe(acac)_3_ Trimethylamine N-oxide Mercaptoethanol β-amyrin	Benzyl ether	None	51.8	262	[71]

UN denotes unknown, as the information was not provided in the literature. SPM denotes superparamagnetic, H_C_ = M_R_ = 0. M_S_ and H_C_ at 300 K. Values in italics indicate that these data were estimated from figures provided in the references.

**Table 4.** Fe_3_O_4_ nanoparticles with special geometries and their magnetic properties.

Morphologies	Size/Dimension (nm)	Magnetic Properties (at 300 K)	Ref.
Nanorods	63 × 6.5 140 × 12	M_S_ = 20.01, H_C_ = 46.53	[79]
Nanorods	41 × 7 65 × 5.7 56 × 10	M_S_ = 86 emu/g M_S_ = 84 emu/g M_S_ = 87 emu/g	[40]
Nanorods	41 × 7	Ms = 86 emu/g, H_C_ = 50 (Oe)	
Nanorods	35 × 5.5 55 × 8 75 × 9 120 × 8 180 × 24	M_S_ = 44 emu/g M_S_ = 53 emu/g M_S_ = 59 emu/g M_S_ = 55 emu/g, H_C_ = 1100 Oe (10K) H_C_ = 850 Oe (10K)	[80]
Nanorods	310 × 135	Superparamagnetic	[81]
Nanoplates	Width (hexagonal): 120 Side length (triangular): 90 Thickness: 7	M_S_ = 84.7 emu/g, H_C_ = 117.72 Oe, M_R_ = 13.36 emu/g	[82]
Triangular nanoprisms	Edge: 113 Thickness: 25	M_S_ = 81.44 emu/g, H_C_ = 126.29 Oe, M_R_ = 11.29 emu/g	[83]
Triangular nanoprisms	Edge: 22 Thickness: 10	UN	[84]
Hexagonal nanoplates	Diameter: 200 Thickness: 20–30	M_S_ = 51.4 emu/g, H_C_ = 263 Oe, M_R_ = 18.9 emu/g	[85]
Multiarmed (bipod, tripod, and tetrapod)	Diameter of arms: 100–200 Length of arms: up to 2 μm	M_S_ = 106.6 emu/g, H_C_ = 148.5 Oe, M_R_ = 30.8 emu/g	[86]
Hollow spheres	Diameter: 16 Shell thickness: 3	UN	[87]
Hollow spheres	Diameter: 120 nm	M_S_ = 85.4 emu/g	[88]
Hollow spheres	Diameter: 295 nm	M_S_ = 76.7 emu/g	[89]
